# ‏Real-time validation of advanced control strategies for grid-connected PV–wind hybrid systems using adaptive starfish and greedy man optimization algorithms

**DOI:** 10.1038/s41598-026-66830-5

**Published:** 2026-08-20

**Authors:** Saad A. Mohamed Abdelwahab, Marwa A. Ali, Wael I. Mohamed, Walid S. E. Abdellatif

**Affiliations:** 1 Department of Electrical, Faculty of Technology and Education, Suez University, P.O.Box: 43221, Suez, Egypt; 2 Department of Basic Science, Higher Institute of Electronic Engineering (HIEE), Belbis, 44621 Egypt; 3 Department of Electrical Engineering, Higher Institute of Engineering and Technology, Kafrelsheikh, 33511 Egypt

**Keywords:** PV wind hybrid system with grid, Adaptive starfish optimization algorithm (ASFOA), Greedy man optimization algorithm (GMOA), Particle swarm optimization algorithm (PSO), Energy science and technology, Engineering

## Abstract

Sophisticated optimization and management techniques are now needed to ensure optimal power harvesting, improved power quality, and reliable grid synchronization due to the rapidly growing demand for renewable systems like wind and photovoltaic (PV) under highly variable environmental conditions. The integration of these resources into smart power grids presents critical challenges related to stability, power quality, and energy extraction efficiency. When applied to a grid-connected hybrid PV–Wind energy system, the Adaptive Starfish Optimization Algorithm (ASFOA), the Greedy Man Optimization Algorithm (GMOA) and the Particle Swarm Optimization Algorithm (PSO) are thoroughly compared in this study with an emphasis on two key optimization layers: Maximum Power Point Tracking (MPPT) and PI controller tuning for inverter control. The MATLAB/Simulink model and simulation of the system include PV arrays, a PMSG-based wind turbine, DC-DC converters, and a PWM-controlled inverter connected to the grid via a stabilized DC-link. The simulation results show that the two approaches perform very differently. GMOA and PSO demonstrated faster convergence, stronger global search capability, and fewer steady-state oscillations, all of which improved DC-link voltage stability and dynamic response under abrupt changes in solar irradiance and wind speed. In contrast, ASFOA achieved higher power accuracy, lower overshoot, and smoother transient response, resulting in reduced power ripples and stronger local optima exploitation. Comparative graphical analysis includes power response curves, convergence profiles, DC-link stability, inverter current waveforms. Comparative graphical analysis including power response curves, convergence profiles, DC-link stability, inverter current waveforms, and Total Harmonic Distortion (THD) spectrum evaluation confirmed the superiority of the optimized controllers over conventional control methods. The system achieved a significant reduction in THD, leading to improved power quality and robust grid synchronization even during abrupt disturbances. This paper is among the first to investigate and validate the combined use of GMOA, PSO and ASFOA in grid-connected hybrid renewable systems, demonstrating that the integration of GMOA for global search and ASFOA for fine local optimization forms a powerful and reliable intelligent control strategy. The proposed approach significantly enhances energy harvesting, power quality, and system resilience, making it a promising solution for real-time energy management and next-generation smart grid applications. Furthermore, the OPAL-RT real-time implementation confirms the robustness and practical feasibility of the proposed ASFOA-based control strategy. The strong agreement between simulation and real-time results demonstrates its effectiveness for real PV systems, providing improved dynamic response and enhanced power extraction under varying environmental conditions.

## Introduction

Renewable and clean energy is becoming more popular worldwide as a means of meeting the growing demand for electricity due to the depletion of conventional energy sources and growing environmental concerns. Solar energy and wind energy are the most dependable renewable energy sources available. However, a drawback of renewable energy generation is its significant reliance on environmental conditions, including temperature, wind speed, solar irradiance, and similar factors, resulting in considerable fluctuations in output characteristics. In this study, wind and solar power generation were combined to account for the output characteristic instability. Maximum power point tracker (MPPT) is used in wind and photovoltaic (PV) generating. The wind solar complementing power supply system is an affordable power source that efficiently uses both solar and wind energy. This kind of power supply system may offer more than simply a good deal in terms of low cost and excellent reliability for some awkward locations. Additionally, wind/solar complementing generation is more economical than either wind or PV power generation alone in terms of cost and energy storage component protection^1^. Effective control and management systems are essential to the correct operation of hybrid micro grids. The literature emphasizes the significance of intelligent control systems that can adjust to shifting conditions, such as load variations, weather variations, and component failures. In-depth dynamic modelling, design, and control of PV and wind as a hybrid system linked to the electrical grid are the main goals of this work. To capture the most power under variable climatic conditions, both solar installations and wind farms use the MPPT extraction technique. This research aims to assess the performance of hybrid systems under different environmental conditions, such as variations in wind speed and sun irradiance^2^.

Conventional PI controllers are often utilized owing to their simplicity, but they lack adaptability under rapidly changing operating conditions, limiting their effectiveness in dynamic environments. Consequently, bio-inspired and evolutionary optimization algorithms such as Genetic Algorithm (GA), Particle Swarm Optimization (PSO), Grey Wolf Optimizer (GWO), SSOA, Ant Colony Optimization (ACO), and more recently Starfish Optimization Algorithm (SFOA) and Greedy Man Optimization Algorithm (GMOA), have emerged as effective solutions for real-time parameter tuning, MPPT enhancement, and improved inverter control. Despite these advancements, key challenges remain insufficiently addressed, including slow convergence in high-dimensional optimization, inability to maintain stable DC-link voltage under sudden weather variations, oscillations around the global MPP, and high inverter current THD^3^.

A significant change in perspective about decentralized and sustainable energy systems is suggested by the body of research on hybrid renewable energy micro grids. Localized energy networks known as micro grids efficiently use a range of renewable energy sources and energy storage technologies to meet fluctuating energy demands. Artificial intelligence techniques, particularly deep learning, have recently gained considerable attention for enhancing the operational efficiency, energy management, and reliability of renewable-based microgrids, highlighting the increasing trend toward intelligent optimization in modern power systems^4–7^. Integrating hybrid renewable energy sources: Micro grid systems must incorporate a range of renewable energy sources, including biomass, hydro, wind, and solar, according to research. The interplay of multiple sources reduces their unpredictability and guarantees a more consistent and dependable energy supply. The best resource combinations are identified by investigations, which take into consideration local variables including resource availability and complementary generation patterns^8–12^.

A few optimization techniques are being studied by researchers for the development and management of effective hybrid renewable energy micro grids. System design optimization, energy generation scheduling, and storage utilization management can all benefit from the application of GA, PSO, and other met heuristic methodologies^13–17^.

The research^18^ introduces an intelligent control strategy for hybrid renewable energy systems connected to the grid. It uses a self-tuning fuzzy MPPT algorithm that automatically adapts to weather changes, permitting the system to extract MPP from both solar and wind sources. This approach improves efficiency, reduces energy losses, and ensures a more stable and consistent supply of clean electricity.

In^19^ presents a hybrid renewable system that integrates solar PV panels with a wind turbine driven by a doubly fed induction generator (DFIG). The study emphasizes an advanced vector control strategy to adjust the active and reactive power of DFIG. This method ensures stable grid connection, maximum power extraction, and improved dynamic performance under variable wind speed and solar radiation. The proposed design enhances system efficiency, reliability, and sustainability, making it suitable for modern clean energy applications.

In^20^, introduces a hybrid renewable system that integrates solar PV and wind energy within a smart grid. The research focuses on improving power quality by reducing issues such as harmonics, voltage fluctuations, and instability. The proposed control method combines inverter-based control with artificial neural network (ANN) techniques, where the ANN intelligently adjusts the inverter operation in real-time to maintain voltage stability, regulate frequency, and optimize power flow. This AI-driven control approach guarantees dependable incorporation of renewable energy sources into the smart grid, improved grid stability, and effective energy management.

In^21^ suggests an operative control approach to enhance the Low-Voltage Ride-Through (LVRT) capability of a PV and wind hybrid power system. The approach modifies the system’s control scheme to improve performance under severe grid faults. The PV boost converter's MPPT function is turned off during faults, enabling the inverter to supply reactive power to maintain voltage stability. To increase the wind farm's LVRT capability, an outside crowbar protection system is simultaneously triggered. The proposed method is tested under voltage sags, symmetrical, and unsymmetrical grid faults, with simulation results confirming important enhancements in the system’s dynamic performance during and after disturbances.

In^22^ investigates PSO-based PI controllers for a grid-connected inverter supplied by renewable energy of hybrid system consisting of wind turbines, PV panels, and two energy storage units. Grid voltage sags and a range of active and reactive power circumstances are simulated for the system and inverter. The findings show that the best dynamic responsiveness and performance are provided by online PSO-optimized PI controllers that are configured according to the ITAE index.

In^23^ presents an intelligent method to improve power quality in grid-connected hybrid systems integrating solar PV and wind energy sources. The study illustrates how important STATCOMs are in reducing power quality problems including voltage swells, sags, and disruptions produced on by nonlinear loads. Simulation results show that renewable energy systems (RES) equipped with STATCOMs perform significantly better than those without. The suggested design efficiently lowers harmonics, minimizes voltage swings, increases surplus power transfer to the grid, and makes better use of renewable energy sources. The GWO algorithm's efficacy in optimizing STATCOM control settings is confirmed by validation using MATLAB/Simulink, indicating its potential as a useful tool for further study and real-world applications in power quality improvement and renewable energy integration.

In^24^, examines the LVRT issue arising from line-to-line faults and its effects on a hybrid system comprising a 9 MW wind farm and a 1 MW PV station. To address this problem, the STATCOM device is employed. The proposed STATCOM is utilized to reduce voltage drop and adjust for reactive power. The PSO optimization method is used with the PID controller to determine the ideal PID parameter. Fuzzy Logic Control (FLC) is superior to the STATCOM PID controller in terms of enhancing power quality and low voltage ride through (LVRT) capacity.

Ref^25^ introduces the study develops and applies an optimization framework for the design of a hybrid power system that combines PV panels, wind turbines, fuel cells and energy storage to meet electrical demand across different Libyan regions. To optimize hybrid renewable energy systems, this study contrasts the Whale Optimization Algorithm (WOA) with the ACO. The findings demonstrate that by reducing the likelihood of power supply failure and raising the percentage of renewable energy, Ant Colony Optimization improves system reliability and RE integration. Consequently, ACO showed excellent system reliability and integration of renewable energy (RE).

In^26^ the study presents an in-depth evaluation of a Hybrid Solar Wind Energy System designed to improve the efficiency and reliability of renewable power generation. The system integrates solar PV and wind energy, both using MPPT techniques to maximize power extraction. A DFIG-based wind system is used for its strong performance under varying wind speeds, while the B2B converter configuration allows separate control of generator and grid operations, enhancing system stability. A significant contribution of the work is the coupling of a standalone solar PV system with both conventional and hybrid MPPT methods. The solar PV subsystem is optimized using the traditional Perturb and Observe (P&O) method and the PSO algorithm, highlighting the value of optimization in improving control effectiveness. The proposed optimized control framework provides several advantages, including reduced system cost by lowering the number of required inverters, better use of real- time solar data, improved DC-link voltage stability under fluctuating irradiance, and strong performance of conventional algorithms within a hybrid configuration.

Ref^27^ this study focuses on a hybrid power system that is connected to the grid and integrates wind and PV energy sources with a hybrid storage system that includes batteries and a supercapacitor. The goal of this research is to reduce total harmonic distortion (THD) fed into the grid while optimizing the energy produced by PV arrays and wind turbines. The COOT bird algorithm was used in this study to optimize a cascade PI-PID controller, and the outcomes were contrasted with those of a GA-tuned PI controller. Using offering a 96% faster settling time and fewer oscillations in current and voltage, the suggested solution, COOT-PI-PID + SC, performed better than the straightforward PI controller optimized using the evolutionary algorithm. This eliminated the PI-PID controller peaks and led to ${\mathrm{T}\mathrm{H}\mathrm{D}}_{\mathrm{i}}$ and ${\mathrm{T}\mathrm{H}\mathrm{D}}_{\mathrm{v}}$ reductions of 30% and 81%, respectively.

The achievement of the recommended method was demonstrated by the 66% reduction in the DC voltage peak undershoot (Us) and the 64% reduction in the peak overshoot. To improve the stability of wind-PV hybrid power systems,^28^ investigates the coordinated optimization of controller parameters of unified power flow controller. To optimize the properties of these controllers, an efficient enhanced Pelican Optimization Algorithm (POA) is created. The suggested method is beneficial in reducing low-frequency oscillations in renewable energy grid integration systems, according to simulation results from hybrid power systems.

Ref^29^ describes GMOA, a new bio-inspired metaheuristic algorithm for dealing with complicated optimization problems. Drawing inspiration from competitive individuals resistant to change, the algorithm introduces two distinctive mechanisms. Its performance on standard benchmark functions is compared to widely used algorithms such as GA, PSO, DE, and ACO. The results show that GMOA achieves superior solution quality, faster convergence, and greater robustness. The importance of these advancements is confirmed by statistical analyses, which also emphasize GMOA's potential for practical uses including supply chain optimization and healthcare resource management, as well as its excellent balance between exploration and exploitation.

In^30^ proposes a novel method to enhance the transient performance of a doubly fed induction generator (DFIG) system using a cascaded PI–PDN controller optimized by the SFOA. To provide better dynamic and steady-state responses, the design uses a customized objective function that combines rising time, settling time, overshoot, and steady-state error. Comparative studies with GA, PSO, GWO, and SSOA algorithms show that the proposed SFOA-tuned controller achieves faster response, reduced overshoot, and higher consistency, demonstrating the effectiveness of SFOA for control system design in renewable energy applications.

In^31^ focuses on the optimal design and performance evaluation of a green hydrogen power-to-power (GHP2P) system for off-grid applications. The proposed configuration integrates PV panels, wind turbines, and a fuel cell with a hydrogen storage tank, enabling efficient chemical energy storage. System component sizing is optimized using two advanced metaheuristic algorithms the SFOA and the Artificial Hummingbird Algorithm (AHA). To assess accuracy, stability, and robustness, both algorithms are tested on three different off-grid GHP2P setups. Simulation results show that AHA consistently outperforms SFOA, achieving the lowest fitness value with shorter computational time. Statistical analysis further confirms its efficiency, highlighting AHA as a highly competitive and promising optimization approach for solving complex engineering design problems.

Recent studies have demonstrated significant progress in applying advanced optimization and intelligent control techniques to grid-connected renewable energy systems. In^32^, an Electric Eel Foraging Optimization (EEFO)-based approach was proposed to improve MPPT performance and PI controller tuning in hybrid PV–wind systems. A robust PI controller based on the Sparrow Search Algorithm (SSA) for grid-connected photovoltaic systems was presented in^33^, demonstrating enhanced dynamic performance in smart microgrid applications. The importance of real-time validation for renewable energy control and energy management strategies was highlighted in^34^ through a real-time simulation testbed. Advanced multi-objective optimization and intelligent energy management strategies for grid-connected hybrid renewable microgrids were further investigated in^35^ and^37^. In addition, advanced control strategies for hybrid renewable energy systems were proposed in^36^ to improve grid stability and power quality under variable operating conditions. Although these studies have reported promising results, most of them focus on a single optimization algorithm, a specific control objective, or energy management applications. In contrast, the present work provides a comprehensive comparative investigation of ASFOA, GMOA and PSO for simultaneous MPPT enhancement and PI controller tuning in a grid-connected PV–wind hybrid energy system, followed by real-time validation using the OPAL-RT platform.

Although many previous studies have focused on developing new MPPT algorithms or improving individual control strategies, the primary objective of this work is different. The proposed ASFOA, GMOA and PSO algorithms are employed to determine the optimal gains of the PI controllers governing the grid-side inverter and the DC-link voltage regulation. Consequently, the optimized controller parameters enhance the overall dynamic performance, grid synchronization, and system stability under varying operating conditions, which in turn leads to improved maximum power extraction from the PV–wind hybrid system. Furthermore, unlike most previous simulation-based studies, the proposed control strategy is comprehensively validated through real-time implementation using the OPAL-RT platform, providing practical evidence of its effectiveness and robustness.

In summary, the study highlights the importance of hybrid renewable energy micro grids in creating resilient and sustainable energy systems. By considering technological developments, optimization strategies, control strategies, and real-world applications, cumulative results show how these micro grids can handle energy challenges and facilitate the shift to decentralized and eco-friendly energy solutions. The main characteristics of the current study are contrasted with a few prior research projects in Table 1.

**Table 1:** compares the characteristics of the current study with earlier research.

Ref	Optimization method	Primary objective	System configuration	Validation method	Main contribution
^18^	Conventional control	MPPT and power regulation	Hybrid PV/Wind	Simulation	Conventional control strategy
^19^	Conventional control	Power management	Hybrid PV/Wind	Simulation	Performance evaluation under varying conditions
^20^	Conventional control	Controller design	Hybrid PV/Wind	Simulation	Conventional control implementation
^21^	Conventional control	MPPT enhancement	Hybrid PV/Wind	Simulation	Improved operating performance
^22^	PSO	PI controller optimization	Hybrid PV/Wind	Simulation	Optimal controller parameter tuning
^23^	GWO	PI controller optimization	Hybrid PV/Wind	Simulation	Intelligent controller optimization
^24^	PSO + FLC	MPPT and intelligent control	Hybrid PV/Wind	Simulation	Hybrid optimization strategy
^25^	WOA + ACO	Energy management	PV/Wind/Fuel Cell/ESS	Simulation	Multi-source energy optimization
^26^	P&O + PSO	MPPT optimization	Hybrid PV/Wind	Simulation	Improved tracking performance
^27^	COOT + GA	Hybrid storage control	PV/Wind + Battery/SC	Simulation	Coordinated storage management
^28^	POA	Controller optimization	Hybrid PV/Wind	Simulation	Optimal control design
^29^	GMOA, GA, PSO, DE, ACO	Optimization algorithm assessment	Conventional system	Simulation	Comparative optimizer evaluation
^30^	SFOA, GA, PSO, GWO, SSOA	Wind turbine control	Wind energy system	Simulation	Intelligent controller tuning
^32^	EEFO	MPPT and PI gain optimization	Grid-connected PV/Wind	Simulation	Joint optimization of MPPT and controller gains
^33^	SSA	Robust PI controller design	Grid-connected PV	Simulation	Robust inverter control
^34^	Energy management strategy	Real-time microgrid control	Renewable microgrid	OPAL-RT	Hardware-in-the-loop validation
^35^	Enhanced MOPSO	Multi-objective energy management	Hybrid renewable microgrid	Simulation	Intelligent energy management
^36^	Advanced control strategies	Grid stability and power quality	Hybrid renewable system	Simulation	Improved dynamic performance
^37^	K-means clustering	Energy management	Grid-connected microgrid	Simulation	Intelligent operational scheduling
This Work	ASFOA & GMOA & PSO	Optimal PI controller gain tuning for enhanced MPPT and grid-connected control	Grid-connected PV–Wind hybrid system	Simulation & OPAL-RT real-time validation	Advanced algorithms with comprehensive real-time verification

The main contribution of the paper is rewritten with more additions which are summarized as follows:

- Under various weather circumstances, the simulation results of the GMOA, PSO and the ASFOA-based MPPT approach for PV–Wind hybrid systems are extracted and presented.
- Assessment Telltale for PV–Wind hybrid efficiency under MPPT strategy is used to demonstrate the feasibility of the ASFOA compared with the GMOA and PSO optimization technique.
- Using the OPAL-RT 4610 platform, the suggested control system's experimental inquiry is carried out in real time. Precise integration of intricate MATLAB/Simulink models is made possible by this sophisticated real-time simulator, enabling exact reproduction of real-world operational conditions.

This paper is arranged as follows, in Section "Introduction" introduction and study contributions, Section "The proposed PV-WIND hybrid with grid", describes the proposed hybrid PV and wind system, Section "Proposed optimization algorithm" delineates the methodologies employed for the proposed algorithms, Section "Results and discussion" displays the hybrid system's simulation results, Section “Proposed system of PV/Wind hybrid system with real-time (OPAL-RT 4610)” and Sect. “Conclusion” concludes the conclusion.

## The proposed PV-WIND hybrid with grid

**Fig. 1:**
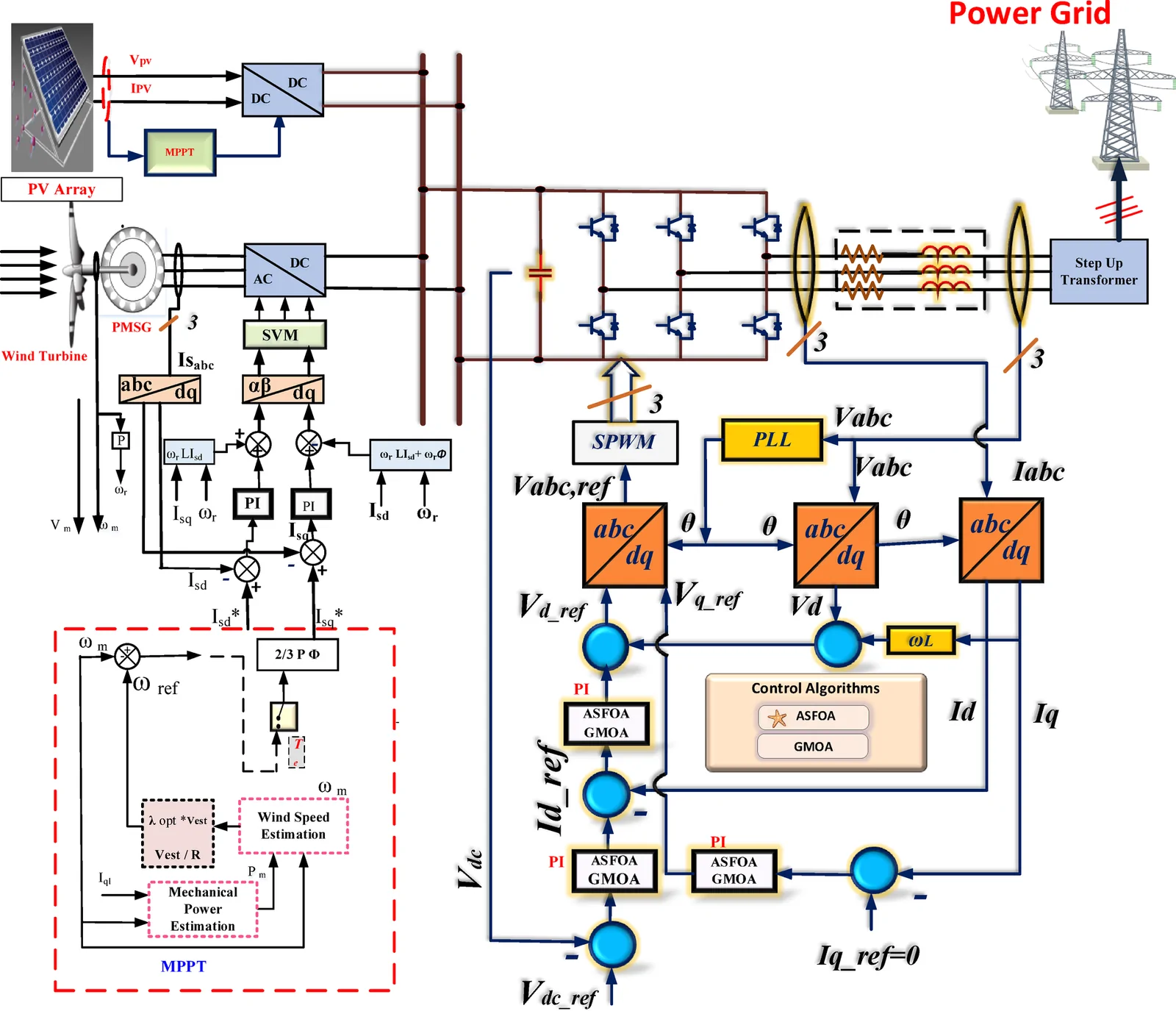
PV- Wind Hybrid System with grid.

To provide dependable and steady power delivery to the utility grid, the suggested hybrid renewable energy system combines PV generation, a wind energy conversion system (WECS) based on a PMSG, and a grid-connected inverter through a unified DC-link structure is shown in Fig. 1. To continually extract the highest amount of solar power possible under changing irradiance and temperature circumstances, the PV subsystem is made up of a PV array and a DC–DC converter managed by an MPPT algorithm. In parallel, the wind subsystem employs a PMSG driven by the wind turbine, where the generator’s variable-frequency AC output is rectified by an AC–DC converter and regulated using ${d}_{q}$-frame current controllers and space vector modulation (SVM).

To achieve maximum aerodynamic energy extraction, the wind MPPT unit estimates the wind speed, tip-speed ratio, mechanical power, and the corresponding optimal generator speed reference. Both renewable sources inject their energy into a common DC-link capacitor, which stabilizes the DC voltage before feeding the grid-side inverter. To provide decoupled control of active and reactive power, the grid-side conversion stage consists of a DC-AC inverter that is synchronized with the electrical grid via a phase-locked loop (PLL). Grid voltages and currents are converted into the ${d}_{q}$-reference frame. While current regulators manage the d-axis and q-axis currents that influence actual and reactive power, voltage regulators maintain the DC-link voltage. Sinusoidal PWM is then used to generate high-quality switching signals for the step-up transformer and final grid injection.

To enhance the overall dynamic performance and ensure optimal coordination between the PV and wind sources, advanced optimization algorithms are embedded within the control architecture. Specifically, ASFOA, GMOA and PSO algorithms intelligently select and tune the optimal PI controller gains for both the voltage and current regulators, ensuring fast convergence, reduced overshoot, and highly stable behavior under environmental fluctuations.

Through this automated gain-selection mechanism, the regulators operate at their most efficient configuration, which significantly improves the stability of the DC-link, enhances power-sharing coordination between the PV and wind system, and ultimately achieves maximum efficiency for grid integration. The additional optimization algorithm further refines power-flow coordination and controller robustness, contributing to high-quality, reliable electricity delivery from the hybrid system.

The hybrid system's grid-tied inverter transports electricity from the PV array, wind turbines and the electric grids. The inverter regulates both the active and reactive energy provided to the grid and the voltage on the DC bus under different climate conditions. The DC voltage is modified using the DC voltage control loop. By introducing zero reactive currents into the utility grid, vector control achieves unity power factor (UPF) operation. Using the recommended control systems, the inner control loops regulate the current direct (${I}_{d}$) and quadrature current (${I}_{q}$). The outer suggested control system loop is used to change the voltage DC to ${V}_{\mathrm{d}\mathrm{c}-\mathrm{r}\mathrm{e}\mathrm{f}}$, the value reference.

The general relationship between grid-tied inverter voltages and line currents can be expressed as follows^38^:

1 $$\left[\begin{array}{c}{E}_{a}\\ {E}_{b}\\ {E}_{c}\end{array}\right]=\left[\begin{array}{c}{V}_{a}\\ {V}_{b}\\ {V}_{c}\end{array}\right]+\left[\begin{array}{c}{I}_{a}\\ {I}_{b}\\ {I}_{c}\end{array}\right]{R}_{f}+\frac{d}{dt}\left[\begin{array}{c}{I}_{a}\\ {I}_{b}\\ {I}_{c}\end{array}\right]*{L}_{f}$$

The inverter output voltages are ${E}_{a}$, ${E}_{b}$, and ${E}_{c}$; The grid three-phase voltages are ${V}_{a}$, ${V}_{b}$, and ${V}_{c}$; the three-phase line current is ${I}_{a}$, ${I}_{b}$, and ${I}_{c}$; and the filter's resistance and inductance are ${L}_{f}$ and ${R}_{f}$, respectively.

The optimization process aims to determine the optimal PI controller gains that achieve fast dynamic response, reduced overshoot, accurate DC-link voltage regulation, and improved maximum extraction while maintaining stable grid-connected operation. The optimized controllers are subsequently validated through both simulation and OPAL-RT real-time implementation.

### PV modeling

**Fig. 2:**
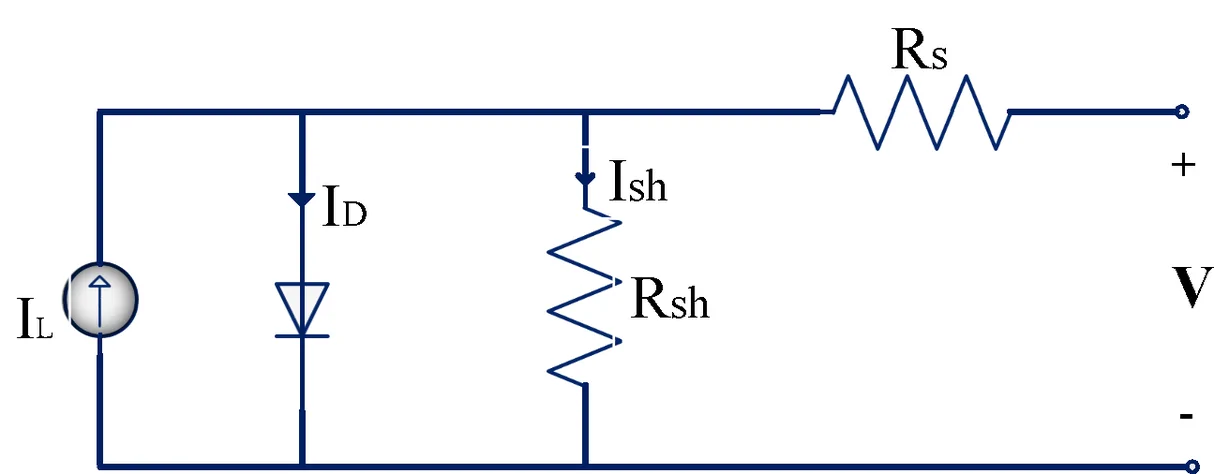
PV cell equivalent circuit.

Understanding and analyzing a solar cell's electrical behavior under different operating conditions requires PV modelling. A current source (${I}_{L}$), a diode, a shunt resistance (${R}_{sh}$), and a series of resistance (${R}_{s}$) make up the equivalent circuit of a PV cell, as seen in Fig. 2. The photocurrent generated by incident sunlight is represented by the current source^38,39^. The diode contributes to the nonlinear voltage-current relationship by compensating for the intrinsic p–n junction characteristics of the PV cell. The ${R}_{sh}$ represents leakage currents at the connection. In contrast, ${R}_{s}$ indicates resistive losses in the cell's material and connections^40,41^. The output current (I) is determined by the balance of these components, following the fundamental PV cell equation^42^. This equivalent circuit serves as the foundation in constructing and optimizing PV-based energy systems, allowing for accurate performance analysis under a variety of irradiance and temperature conditions^43,44^.

2 $${\mathrm{I}}_{D}={I}_{0} \left\{\mathrm{exp}\left[\mathrm{A}\mathrm{p}\mathrm{v} \left({\mathrm{V}}_{PV}+{\mathrm{I}}_{PV}*{R}_{s}\right)\right]-1\right\}$$

3 $${\mathrm{I}}_{PV}={\mathrm{I}}_{L}-{\mathrm{I}}_{D}-{\mathrm{I}}_{RSh}$$

4 $${V}_{PV}= - {R}_{s }{I}_{PV}+ \frac{1}{{A}_{g}} \mathrm{L}\mathrm{n} \left(1+ \frac{{I}_{L}* \varphi *I}{{I}_{S}*{10}^{-8}}\right)$$

A calibration procedure that comprised curve fitting the single-diode model to experimental I-V data under typical testing settings was used to estimate the PV module specifications. By minimizing the difference between simulated and measured output characteristics, the reverse saturation current (${I}_{s}$), series resistance (${R}_{s}$), the normalized solar irradiance coefficient (*φ *) and diode ideality factor (A_g_) were calculated. The short-circuit current at 100% insolation is represented by the photocurrent (${I}_{L}$), which has been modified according to temperature and irradiance level. This parameter identification guarantees an accurate description of the PV module's electrical behavior for further modeling and performance evaluation^45,46^.

### Wind turbine modeling

The PMSG's high efficiency, small size, and superior dynamic performance make it a popular component of contemporary wind energy conversion systems. In contrast to traditional generators, the PMSG reduces electrical losses and eliminates the need for external stimulation by using permanent magnets on the rotor to produce a steady magnetic field. This feature allows the generator to run efficiently at varying wind speeds while maintaining consistent power output. The PMSG outputs variable-frequency AC power, which is subsequently rectified and processed through ${d}_{q}$-frame current control to ensure smooth power flow to the DC-link. Its robust design reduced maintenance requirements, and superior energy-conversion capability make the PMSG a highly suitable choice for hybrid renewable energy systems that integrate wind power with PV generation and grid-connected inverters.

The following relationships can be used to express the wind turbine's mechanical torque and mechanical power^47^:

5 $${P}_{m}=0.5 \pi {.R}^{2}.\rho .A.{\upsilon }^{3}.{C}_{P}(\lambda ,\beta )$$

6 $${T}_{m}=\frac{{P}_{m}}{{\omega}_{m}}$$

${T}_{m}$ stands for mechanical torque (N.m), ${P}_{m}$ for mechanical power (W), R for blade length (m), ρ for wind turbine air density (kg/m3), *υ * for wind turbine speed (m/s), β for blade pitch angle (deg), ${C}_{P}$ for power coefficient, λ for TSR, and ${\upomega}_{m}$ for wind turbine rotational speed (rad/s). The pitch angle (β) and TSR (λ) of the blades determine the ${C}_{P}$. TSR of WT can be written as follows^48^:

7 $$\lambda =\frac{{\omega}_{m} R}{{\upsilon}_{m}}$$

**Fig. 3:**
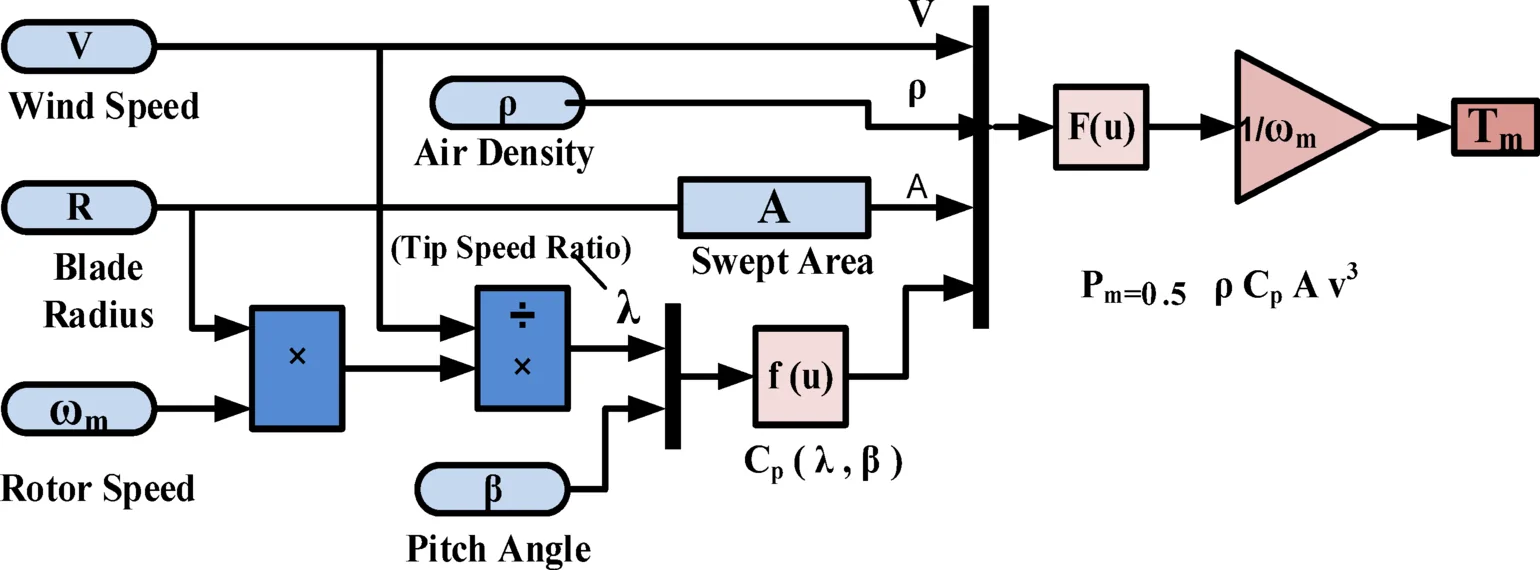
Wind turbine block diagram.

By using the equations^5,6^, it is possible to build the block diagram of the wind turbine as represented in Fig. 3.

**Fig. 4:**
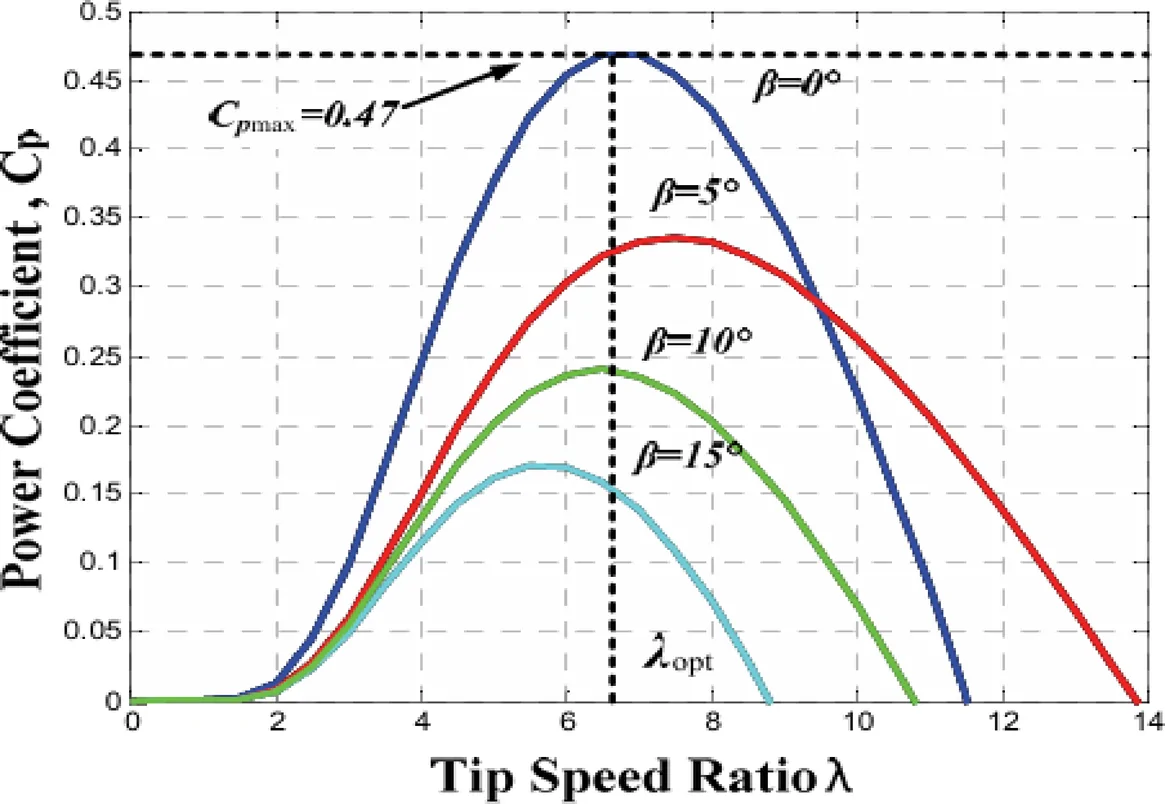
${C}_{P}$ curves as function of ${\lambda }$ and β.

The power coefficient ${C}_{P}$ is one factor that converts wind kinetic energy into mechanical rotational energy. This coefficient is typically calculated using each turbine's experimental curves. A mathematical model has been developed to obtain a generalized from of the coefficient presented in (8) and (9) a set of proposed values for the coefficients ${C}_{1}$ through ${C}_{6}$^49^. Figure 4 shows a typical set of ${C}_{P}$ curves as a function of the linear speed ratio for different pitch angle values for turbine^50^:

8 $${C}_{P}\left(\lambda ,\beta \right)= {C}_{1} \left(\frac{{C}_{2}}{{\lambda}_{1}}- {C}_{3} \beta -{C}_{4} \right){e}^{\frac{{-C}_{5}}{{\lambda}_{1}}} + {C}_{6} \lambda $$

9 $$\frac{1}{{\lambda}_{1}}= \frac{1}{\lambda -0.089 \beta }- \frac{0.035}{{\beta }^{3}+1} $$

The mechanical power on the turbine axis seems to be directly proportional to ${C}_{P}$ in Eq. (5).

Figure 4 illustrates the link between a wind turbine's power coefficient (${C}_{P}$) and tip speed ratio (λ) at various blade pitch angle (β) values. The power coefficient, which is the ratio of the mechanical power extracted to the total wind power available, is a measure of the turbine's aerodynamic efficiency. The figure illustrates that at the ideal tip speed ratio ${\lambda}_{opt}$ ≈ 7, ${C}_{Pmax}$ ≈ 0.47 happens, and the maximum power coefficient is reached when the pitch angle is β = 0°. At this operating point, the turbine blades interact with the airflow at an optimal angle of attack, enabling maximum aerodynamic lift and minimal drag, which leads to the highest possible energy extraction. These ${C}_{P}$ characteristics highlight the importance of operating the turbine around the ${\lambda}_{opt}$ to ensure MPP extraction. This concept is fundamental in designing and implementing MPPT strategies for wind energy conversion systems, including those based on PMSGs.

A synchronous rotating reference frame with the q-axis 90˚ ahead and the d-axis aligned with the rotor flow direction has been used to display a dynamic model of PMSG. In this reference frame^51–53^, the PMSG mathematical equations are described as follows:

10 $$\left|\begin{array}{c}{v}_{sd}\\ {v}_{sq}\end{array}\right|= -{R}_{s} \left|\begin{array}{c}{i}_{sd}\\ {i}_{sq}\end{array}\right|- p\left|\begin{array}{c}{\Psi}_{sd}\\ {\Psi}_{sq}\end{array}\right|{\omega}_{e} \left|\begin{array}{c}{\Psi}_{sq}\\ {\Psi}_{sd}\end{array}\right|$$

11 $${\Psi}_{sq}{ =L}_{d} {i}_{sd}- {\Psi}_{PM}$$

12 $${\Psi}_{sq}= {L}_{q} {i}_{sq} $$

After substituting Eqs. (11,12) into (10) and converting, the next form is achieved:

13 $$\mathrm{P}\left|\begin{array}{c}{v}_{sd}\\ {v}_{sq}\end{array}\right|= -{R}_{s} \langle \begin{array}{c}- \frac{{R}_{s}}{{L}_{d}}\\ -\frac{{R}_{s}}{{L}_{q}}\end{array} |\begin{array}{c}{\omega}_{e} \frac{{L}_{q}}{{L}_{d}}\\ -{\omega}_{e} \frac{{L}_{d}}{{L}_{q}}\end{array} \rangle \left|\begin{array}{c}{i}_{sd}\\ {i}_{sq}\end{array}\right|+ {\omega}_{e} \left|\begin{array}{c}0\\ {\Psi}_{PM}\end{array}\right|- \left|\begin{array}{c}\frac{{v}_{sd}}{{L}_{d}}\\ \frac{{v}_{sq}}{{L}_{q}}\end{array}\right|$$

where ${R}_{s}$ is the stator resistance, ${i}_{sd}$ and ${i}_{sq}$ are the components of the stator current vector in the d and q axes, and ${v}_{sd}$ and ${v}_{sq}$ are the elements of the stator voltage vector in the d and q axes. The direct and quadrature vector components of the stator flux connections in the d and q axes are denoted by ${\Psi}_{sd}$ and ${\Psi}_{sq}$ the direct and quadrature stator inductances are denoted by ${L}_{d}$ and ${L}_{q}$. P = d/dt is the differential operator; np is the number of PMSG pole pairs; and ${\Psi}_{PM}$ is the flux linkage created by the permanent magnets. The PMSG rotor's electrical and mechanical angular speeds, denoted by ${\omega}_{e}$ and ${\omega}_{m}$, are as follows:

14 $${\omega}_{e}={n}_{p}.{\omega}_{m}$$

The generator's electromagnetic torque can be written as follows:

15 $${T}_{e}= \frac{3}{2} {n}_{p}. \left[{\Psi}_{PM} {i}_{sd}- \left({L}_{d}- {l}_{q}\right){i}_{sd} {i}_{sq}\right]$$

For a non-salient-pole machine, the stator direct and quadrature inductances, ${L}_{d}$ and ${l}_{q}$, are roughly equal.

The equation for electromagnetic torque can be described as follows:

16 $${T}_{e}= \frac{3}{2} {n}_{p} {\Psi}_{PM} {i}_{sq}$$

The wind turbine system's mechanical motion equation can be represented as:

17 $${T}_{t}- {T}_{e}=J. \frac{d}{dt} {\omega}_{m}+ {B}_{f} {\omega}_{m}$$

where ${B}_{f}$ is the coefficient of viscous friction and J is the total moment of inertia.

## Proposed optimization algorithm

### Particle swarm optimization (PSO)

Inspired by the collective behavior of biological swarms, Particle Swarm Optimization (PSO) is a metaheuristic method. In this paradigm, particles moving across a (n-dimensional) search space are used to represent potential solutions. Both individual learning and swarm social interaction have an impact on the stochastic velocity updates that control these particles' paths^54,55^.

PSO functions in either global (*gbest*) or localized (*lbest*) configurations, depending on the population's topological connection. Each particle in the global variant can modify its velocity according to its previous best position (${P}_{best}$) and the globally optimal candidate (${G}_{best}$) since the entire swarm exchanges information. On the other hand, the local variation improves local exploitation and reduces premature convergence by limiting information flow to restricted regions (${N}_{best}$).

The dynamic behavior of particle *i* at iteration *k* is characterized by updating its velocity vector (${V}_{ i}^{k+1}$) and position vector (${X}_{ i}^{k-1}$) according to Eqs. (18) and (19).

18 $${V}_{ i}^{k+1}=W{V}_{i}^{k}+{C}_{1} {r}_{1}\left({P}_{best}-{X}_{i}^{k}\right)+{C}_{2} {r}_{2} \left({G}_{best}-{X}_{i}^{k}\right)$$

19 $${X}_{ i}^{k-1}={X}_{i}^{k}+ {V}_{ i}^{k+1}$$

where *W* represents the inertia weight balancing exploration and exploitation; ${C}_{1}$ and ${C}_{2}$ denote the cognitive and social acceleration coefficients, respectively; and ${r}_{1}$ ${r}_{2}$ ~ U (0, 1) are uniformly distributed stochastic variables introducing variance into the search process^56,57^.

### Greedy man optimization algorithm (GMOA)

The proposed study introduces the GMOA as a novel optimization approach for PV–Wind hybrid systems, with the main objective of enhancing MPPT and inverter PI control under varying operating conditions. Unlike conventional methods that often focus on single subsystems, GMOA is uniquely applied to the integrated hybrid system, leveraging its biologically inspired exploration balance to achieve more adaptive and robust control. This contributes to maximizing power extraction, improving system reliability, and ensuring efficient grid integration, while also providing valuable insights into the comparative strengths of GMOA relative to other optimization techniques in renewable energy applications^58–60^.

The behavior of a greedy man dealing with resistant parasites served as the motivation for the GMOA, a population-based metaheuristic. In contrast to traditional algorithms, GMOA strikes a balance between exploration where resistant parasites contribute unpredictability to avoid local optima and exploitation where solutions are greedily enhanced by adopting better neighbors. This approach guarantees enough diversity to prevent early stagnation as well as convergence toward high-quality solutions.

Initializing a population of potential solutions between predetermined lower and higher boundaries is the first step in the mathematical formulation of GMOA. An objective function is used to assess each solution. While the resistance factor probabilistically precludes direct replacement, the greedy selection process decides if an adjacent solution dominates the present one. To further enhance inquiry, periodic incorporation of mutation tactics inspired by parasite eradication is also made. The whole mathematical model of GMOA, including initialization, fitness assessment, dominance comparison, parasite resistance, and mutation operators, is shown in the following equations. To guarantee clarity and repeatability, definitions of all variables and parameters are given.

The initial population of candidate solutions is generated as^61–63^:

20 $${X}_{i}^{0}=LB+rand\left(1,d\right)\times \left(UB-LB\right), i=\mathrm{1,2},\dots .,N$$

where, (${X}_{i}^{0}$) initial position of the ${i}^{th}$ solution, (*LB* ) lower bound vector ${[X}_{1}^{L}, {X}_{2}^{L}, \dots \dots ..{X}_{d}^{L}]$, (*UB* ) upper bound vector ${[X}_{1}^{U}, {X}_{2}^{U}, \dots \dots ..{X}_{d}^{U}]$, (*d* ) dimension of the issue, (*N*) population size, rand $\left(1,d\right)$ = uniformly distributed random vector in [0,1].

Each candidate solution is evaluated as:

21 $$F\left({X}_{i}\right)=f\left({X}_{i}\right)+\uplambda \cdot \mathrm{V}({X}_{i})$$

where, $F\left({X}_{i}\right)$ Penalized objective function value of solution *i*, $f\left({X}_{i}\right)$ Original objective function (s), *λ * penalty coefficient (large positive constant), $\mathrm{V}\left({X}_{i}\right)$ Constraint violation measure.

For each candidate, a random neighbor ${X}_{j}$​ is selected and compared:

22 $${X}_{i}^{t+1}=\left\{\begin{array}{c}{X}_{j}^{t} if \left({X}_{j}^{t}\right)\prec F\left({X}_{i}^{t}\right)and rand >{r}_{i}\\ {X}_{i}^{t} otherwise\end{array}\right.$$

where, (${X}_{i}^{t+1}$ ) updated position of solution iii at iteration *t+1*, ($f({X}_{j}^{t})$≺$f({X}_{i}^{t})$ ) neighbor dominates current solution, (*rand* ) random number in $[\mathrm{0,1}]$, (${r}_{i}$) resistance factor of solution ${\boldsymbol{i}}$, ${r}_{i}\in (\mathrm{0,1})$

At certain intervals, solutions are perturbed to increase diversity:

23 $${X}_{i}^{t+1}={X}_{i}^{t}+\upsigma . \left(\mathrm{U}\mathrm{B}-\mathrm{L}\mathrm{B}\right). N(\mathrm{0,1})$$

where, (*σ * ) mutation scaling factor (small, e.g., 0.01), $N(\mathrm{0,1})$ standard Gaussian random variable, ( $\mathrm{U}\mathrm{B}-\mathrm{L}\mathrm{B}$ ) search space range.

The non-dominated set is updated as:

24 $${A}^{t+1}=\{X\in ({A}^{t}\cup {P}^{t})\mid \nexists Y:F\left(Y\right)\prec F\left(X\right)\}$$

where, (${A}^{t}$) Pareto archive at iteration *t*, (${P}^{t}$) current population at iteration *t*, [$F\left(Y\right)\prec F\left(X\right)$] solution YYY dominates solution *X*

When a convergence requirement is satisfied or the maximum number of iterations is reached, the process ends:

25 $$t\ge {t}_{max} or \mid {F}_{best}^{t}-{F}_{best}^{t-1}\mid <\upepsilon $$

where, (${t}_{max}$​) maximum number of iterations, (${F}_{best}^{t}$) best objective value at iteration *t*, (*ε *) convergence tolerance.

**Fig. 5:**
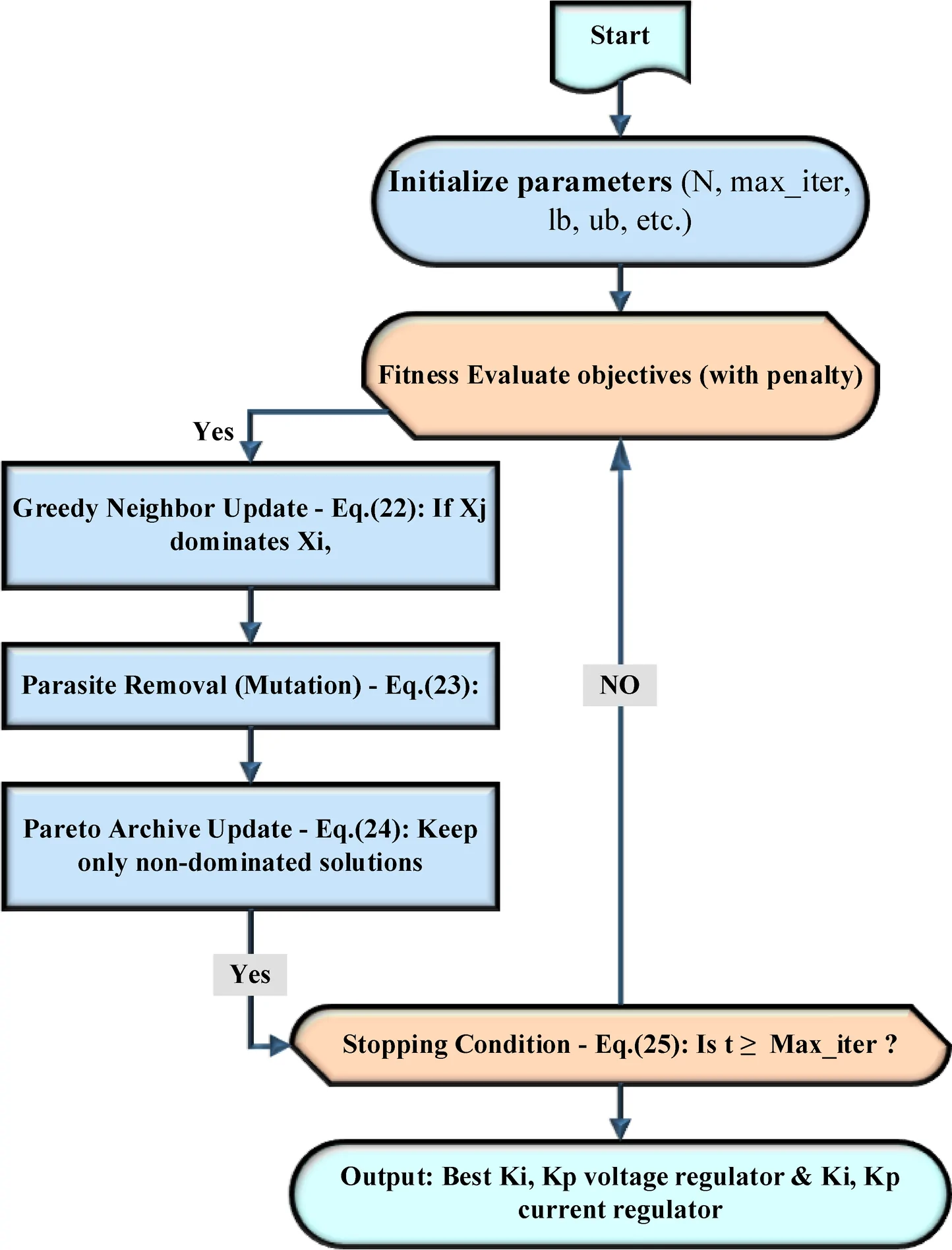
Operational flow diagram of GMOA used in hybrid PV–Wind control optimization.

The flowchart outlines the computational steps of the proposed GMOA, which is applied for optimal control within the hybrid PV/Wind system as displayed in Fig. 5.

Starting with parameter initialization and generation of the initial population (Eq. 20), the algorithm evaluates candidate solutions using the penalized fitness function (Eq. 21). Iterative enhancement is achieved through greedy neighbor selection with resistance (Eq. 22), while mutation is introduced (Eq. 23) to improve robustness under fluctuating renewable inputs. The Pareto archive (Eq. 24) maintains non-dominated solutions relevant to MPPT and inverter PI control. The process terminates when the convergence condition is satisfied (Eq. 25), yielding optimized control parameters that enhance power extraction from PV and wind sources, regulate inverter performance, and ensure high-quality current injection into the utility grid.

### Adaptive starfish optimization algorithm (ASFOA).

ASFOA is a population-based metaheuristic optimization algorithm inspired by the foraging behavior of starfish^64^. In this algorithm, a group of starfish (representing potential solutions) collaboratively search for the optimal solution by adjusting their positions in the search space. The algorithm mimics the decentralized nature of starfish foraging, where everyone makes decisions based on local information and communicates with neighboring starfish to collectively explore the search space^65^.

The ASFOA was initially put forth by artificial intelligence researchers as a novel method for resolving challenging optimization issues. Since its inception, the algorithm has undergone several iterations and improvements to enhance its performance and efficiency^66^. Researchers have conducted extensive studies to understand the underlying principles of starfish foraging behavior and how it can be effectively translated into a computational optimization algorithm. Through these efforts, the ASFOA has emerged as a powerful tool for solving a wide range of optimization problems in various fields, including engineering, finance, and bioinformatics^67^.

One key advantage of the ASFOA is its ability to adapt and evolve its search strategy based on the characteristics of the problem at hand^68^. The ASFOA can swiftly adapt to shifting conditions and discover the best answers by constantly modifying its foraging behavior and investigating various areas of the search space. This adaptability is particularly useful in dynamic optimization problems where the optimal solution may change over time. Furthermore, the scalability of the ASFOA allows it to handle large-scale optimization problems with ease, making it a valuable tool for tackling real-world challenges in various industries^69^.

The ASFOA operates by simulating the foraging behavior of social insects, such as ants or bees, to efficiently search for optimal solutions. Everyone in the population represents a potential solution to the problem, and the algorithm iteratively updates these solutions based on their fitness and the information gathered from other individuals^70^.

Through the process of communication and collaboration, the ASFOA can effectively explore the search space and converge towards the best possible solution. This cooperative nature of the algorithm allows it to outperform traditional optimization techniques in complex and dynamic environments^71^.

**Fig. 6:**
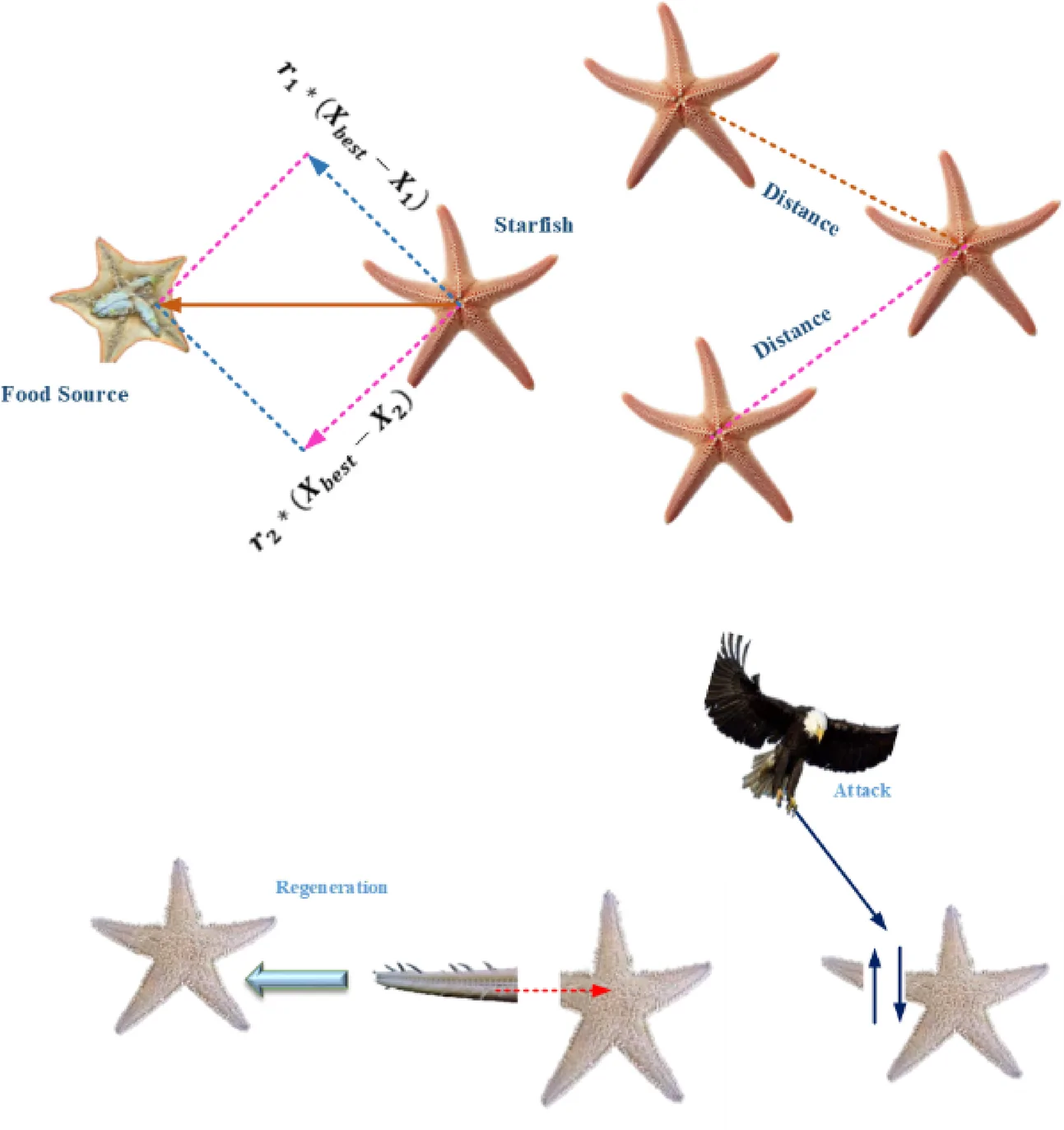
Conceptual illustration of the ASFOA hunting behavior, distance-based movement, threat response, and arm regeneration mechanism.

Sea stars, also referred to as starfish, are marine invertebrates that belong to the Asteroidean class of echinoderms. Starfish, of which there are about 2000 types in the globe, are distinguished by their star-like appearance due to their five arms and central disc. Some kinds of starfish can have seven or more limbs. Their sizes, which are usually between 12 and 25 cm, vary from 0.5 mm to more than 50 cm. Because they lack an osmoregulation mechanism, starfish are rare in freshwater habitats and can be found across the world's oceans, especially in the deep sea. Some starfish can live up to 35 years, although the majority only live for around ten. Several images of starfish are shown in Fig. 6^72,73^.

Figure 6 illustrates the main behavioral principles of the ASFOA. In the first stage, a starfish moves toward a food source (optimal solution) by estimating distances between its current position and the best-known solution. The movement direction is determined using selected coordinate vectors to improve exploration of the search space. In the second stage, the starfish interact with other individuals and update positions based on their relative distances, enhancing search diversity.

Finally, when facing a threat, the starfish initiates arm detachment and regeneration, which is mathematically modeled as a regeneration mechanism to escape local minimum and improve global convergence. These procedures aid ASFOA in optimizing while preserving a balance between exploration and exploitation.

The mathematical formulation of the ASFOA involves defining the fitness function, the population of solutions, and the communication and update mechanisms. By formalizing these components, researchers can analyze the algorithm's convergence properties and performance characteristics. This rigorous approach ensures that the ASFOA is not only effective in practice but also grounded in theoretical principles^74^.

Its application to optimization issues is motivated by mimicking starfish hunting, exploring, and regeneration cycles. In the ASFOA exploration phase, the search method combines a unidimensional search in the case of D < 5 and a five-dimensional search strategy in dimension (D > 5). The five eyes on the starfish's limbs are used to conclude the size threshold. When the optimization issue has more than five dimensions, the search space gets very large. To efficiently scan the environment in such circumstances, the starfish need to use all five arms. It is necessary to use the most well-known position information among the persons to direct the movement of these arms. Equation 26 is then used to update their positions.

26 $${Y}_{i,p}^{T}=\left\{\begin{array}{c}{X}_{i,p}^{T}+\alpha 1*\left({X}_{best,p}^{T}-{X}_{i,p}^{T}\right)*COS \theta , r\le 0.5\\ {X}_{i,p}^{T}-\alpha 1*\left({X}_{best,p}^{T}-{X}_{i,p}^{T}\right)*Sin\theta, r>0.5\end{array}\right.$$

where ${Y}_{i,p}^{T}$ and ${X}_{i,p}^{T}$ denote a starfish obtained and current positions, respectively. ${X}_{best,p}^{T}$ represents the *p*-dimensional best position, where p is the collection of five dimensions chosen at random from the D-dimensional space. A random number between 0 and 1 is called r.

27 $$\alpha 1= (2r-1)*\pi $$

28 $$\theta=\frac{\pi }{2}*\frac{T}{{T}_{max}}$$

*α 1* And *θ* are calculated with Eq. (27) and Eq. (28). *T* Symbolize the existing iteration, and ${T}_{max}$ signifies the maximum iteration. The sine and cosine terms indicate that the starfish’s arms can turn left or right to approach the food with the same probability. *α 1* is randomly generated for each candidate, ensures that the location is updated during the exploration phase, and θ is replaced by the number of iterations, where $\theta \in [0, \pi /2].$ These two parameters help to calculate the influence of the distance between the best and current locations.

It updates the exploration phase's position using the one-dimensional search technique when the optimization issue has fewer than five dimensions. The starfish uses the position data from the other population to guide only one arm to the food source. Equation (29) expresses the revised starfish location.

29 $${Y}_{i,p}^{T}={E}_{t}*{X}_{i,p}^{T}+{A}_{1}*\left({X}_{K1,p}^{T}-{X}_{i,p}^{T}\right)+{A}_{2}*\left({X}_{K2,p}^{T}-{X}_{i,p}^{T}\right)$$

30 $${E}_{t}=\frac{{T}_{max}-T}{{T}_{max}}*COS\theta$$

${X}_{K1,p}^{T}$ and ${X}_{K2,p}^{T}$ are the *p* dimensional positions of 2 randomly designated starfish from the population. A1 and A2 are 2 random numbers within the range (− 1, 1). *p* is a randomly designated dimension from the *D* dimensions, and *Et* denotes the energy of the starfish, formulated in Eq. (30).

To identify global solutions, two update mechanisms are used in the exploitation phase to develop hunting and regeneration behaviors in ASFOA. To replicate the hunting phase of the *i*-th starfish, this phase employs a two-way search scheme that creates use of data from other starfish as well as the position of the best individual in the population. Initially, the distance between five randomly chosen starfish from the population and the ideal position is computed as follows:

31 $${d}_{m}=\left({X}_{best}^{T}-{X}_{{m}_{p}}^{T}\right),m=1,\dots \dots .,5$$

32 $${Y}_{i}^{T}={X}_{i}^{T}+{r}_{1}*{d}_{m1}+{r}_{2}*{d}_{m2}$$

With ${m}_{p}$ representing the five randomly chosen starfish, Eq. (31) yields the distance between the global best and the other five starfish. Equation (32) models each starfish's update rule in its hunting behavior. The random numbers ${r}_{1}$ and ${r}_{2}$ fall between 0 and 1. Among the ${d}_{m}$, ${d}_{m1}$ and ${d}_{m2}$ are chosen at random. While other candidates tend to travel backwards in the same iteration, starfish candidates use the two-way search approach to guide movement toward the best answer. Thus, in the same way, candidates aid in solving the local optimal problem. Because they move slowly when hunting, starfish are especially susceptible to predators, a starfish may lose an arm to get away if a predator spots it. Slow movement is a natural feature of their nature, even though it could be interpreted as vulnerability. When *i* is *N*, the regeneration phase of the ASFOA specifically targets the population's final member. The starfish move slowly because it takes several months for the regeneration process to complete. The position and the regeneration stage rule are updated by Eq. (33) as well.

33 $${Y}_{i}^{T}=\mathrm{exp}\left(-\frac{T*N}{{T}_{max}}\right)*{X}_{i}^{T}$$

where *T*, ${T}_{max}$, and *N* stand for the population size, the maximum number of iterations, and the current iteration, respectively. The exploitation and regeneration steps are combined, and each repetition only involves one calculation. To prevent local solutions and enhance the ASFOA's global convergence, this phase is essential^75^.

**Fig. 7:**
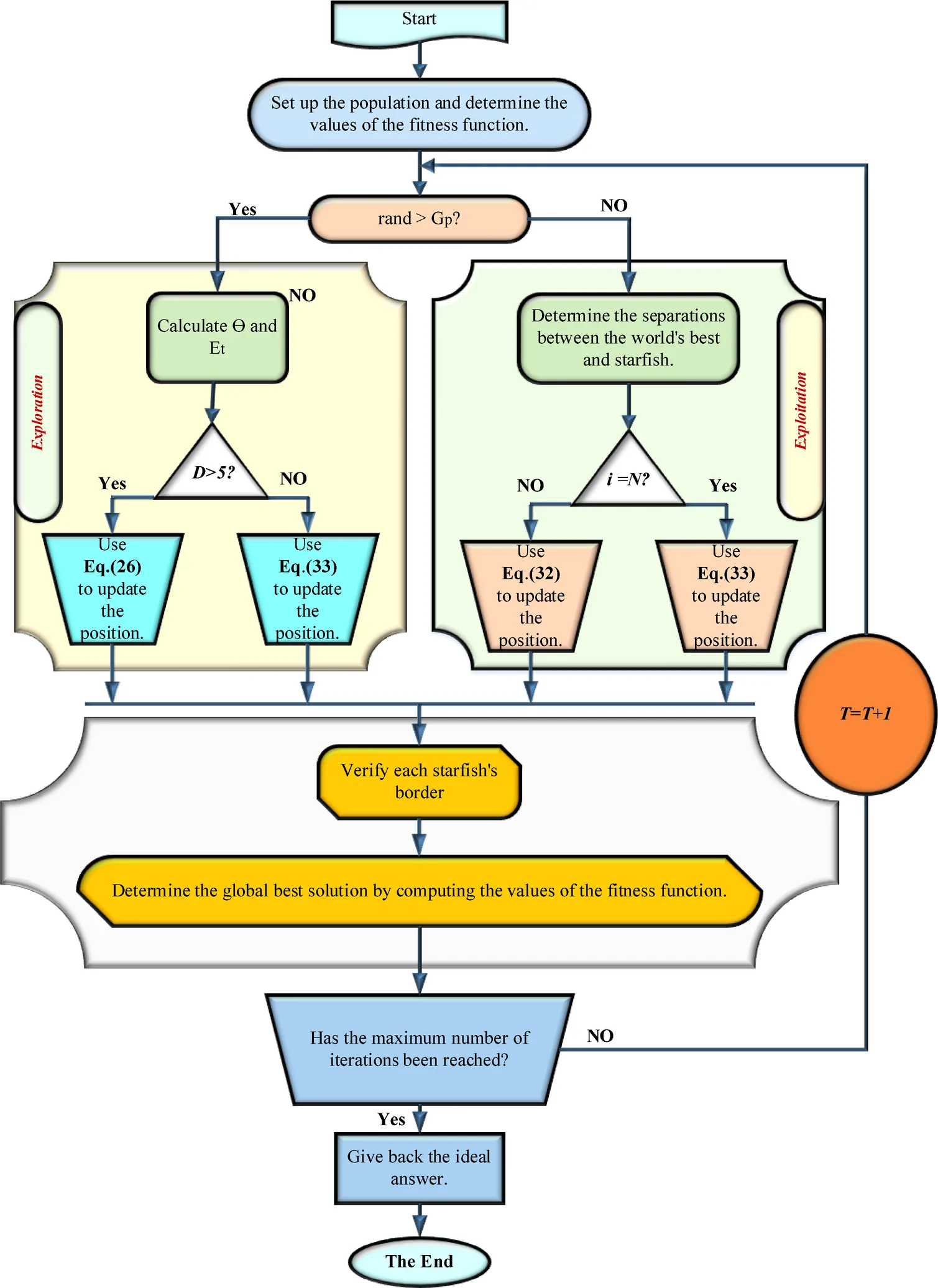
Flowchart of the ASFOA for gain tuning (${K}_{P}$, ${K}_{i}$) in hybrid PV–Wind systems.

As shown in Fig. 7, the flowchart describes the computational stages of the suggested ASFOA, which is used for optimal control within the hybrid PV/Wind system.

## Results and discussion

### Ramp profile for solar irradiance and step profile for temperature and wind speed

Table 2 summarizes the proposed grid-connected hybrid PV–Wind system parameters, including the electrical characteristics of the PV array, wind system, DC-link, grid interface, and control strategy. The PV subsystem is rated at 250 kW and ensuring an appropriate DC voltage level for grid integration.

**Table 2:** System parameters of the proposed PV–wind grid-connected configuration.

Subsystem	Parameter	Value
PV system	PV array power	250 kW
	converter inductance	5 × 10⁻^3^ H
	Converter resistance	0.005 Ω
	converter capacitance	100 × 10⁻⁶ F
Wind system	Wind power (P_wind_)	80 kW
	Rectifier resistance	100 Ω
	Rectifier capacitance	0.1 × 10⁻⁶ F
	Wind speed operating range	7–9 m/s
DC-link	Nominal DC-link voltage	260 V
	DC-link voltage ripple limit	± 5%
Grid interface	Step-up transformer rating	260 V / 25 kV
	Grid voltage	20 kV
	Grid frequency	60 Hz
	Rated grid-injected power	150 kW
Control and optimization	Optimization Technique	ASFOA / GMOA/ PSO
	Operating mode	Grid-connected
	Environmental profiles	Ramp, Step, Random
PI control	ASFOA for voltage regulators	KP = 6.7936
		KI = 987.1911
	ASFOA for current regulators	KP = 0.33748
		KI = 29.4863
	GMOA for voltage regulators	KP = 8.278703
		KI = 882.418435
	GMOA for current regulators	KP = 0.344417
		KI = 18.206583
	PSO for voltage regulators	KP = 7.7438
		KI = 894.2311
	PSO for current regulators	KP = 0.33571
		KI = 23.4321

The selected converter parameters are optimized to reduce current and voltage ripples under dynamic operating conditions.

For consistency and fairness, both ASFOA, GMOA and PSO were executed under identical optimization settings. The population size was set at 50, and the maximum number of iterations was fixed at 100. These parameters were selected after preliminary tuning to achieve a compromise between solution quality, convergence speed, and computational cost.

The wind energy subsystem is rated at 80 kW and operates within a wind speed (WS) range of 8 to 9.5 m/s, representing realistic and variable environmental conditions. The DC-link is regulated at a nominal voltage of 260 V with a strict ripple limit of ± 5%, enhancing system stability. Grid integration is achieved through a 260 V / 25 kV step-up transformer, into a 20 kV, 60 Hz grid.

The table presents the PI controller parameters tuned using ASFOA, GMOA and PSO and for both voltage and current regulation loops. The ASFOA-tuned controllers exhibit balanced proportional and integral gains, contributing to faster dynamic response and improved stability compared to the GMOA and PSO tuned counterparts. The parameter set establishes a realistic and robust framework for evaluating the performance of the suggested optimization techniques under ramp, step, and random environmental profiles.

**Fig. 8:**
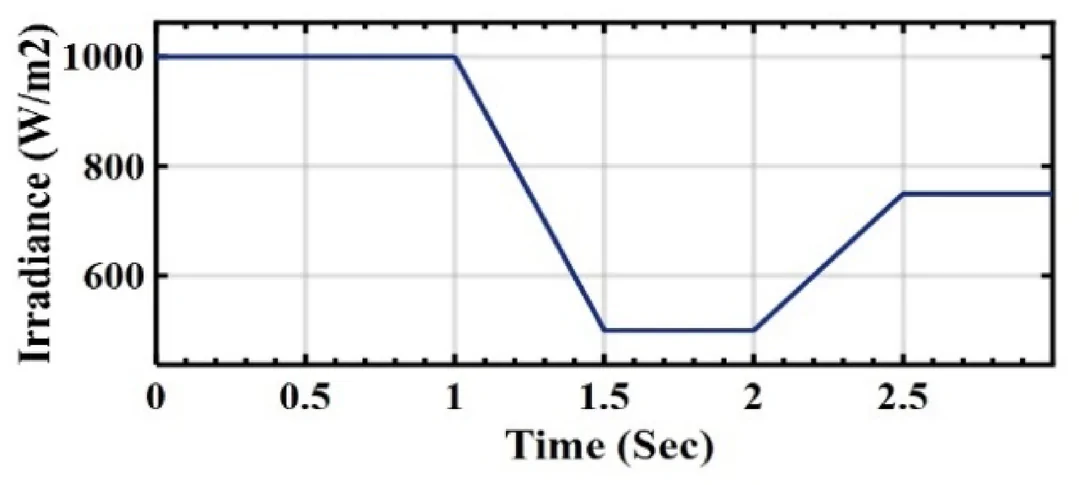
Ramp profile of solar irradiance applied to the PV system.

**Fig. 9:**
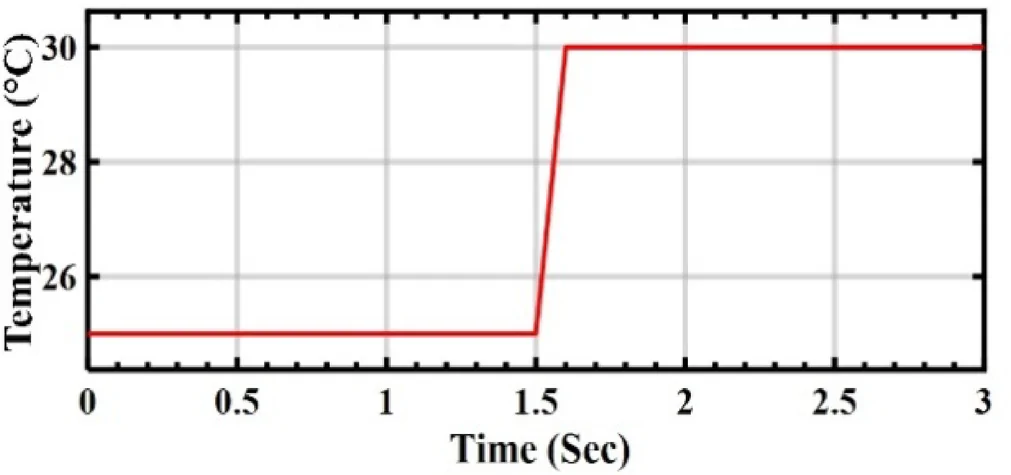
Step change in PV temperature profile.

**Fig. 10:**
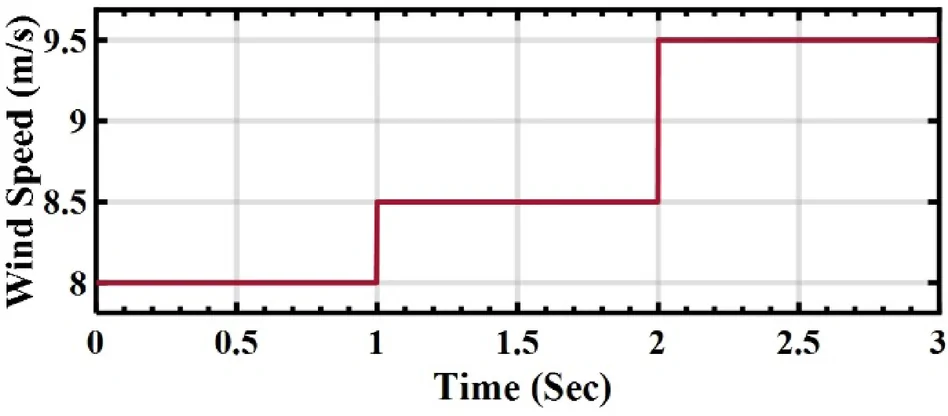
Wind speed step change profile applied to the wind turbine.

To estimate the dynamic act of the hybrid PV–Wind system under realistic environmental variations, a ramp varying profile for solar irradiance and step-change profiles for both temperature and wind speed were applied as displayed in Fig. 8 ,9, and 10.

Figure 8 shows the solar radiation profile which changes gradually. It starts at 1000 W/m^2^ and finds stability at this point in between 0 and 1 s. Then, its value drops to 500 W/m^2^ from 1 s to 1.5 s, and the radiation starts to stabilize during the time from 1.5 to 2 s and increasing from 500 to 750 W/m^2^ during the time from 2 to 2.5 s. These variations in radiation have a significant impact on the current and voltage, and power output for the solar power plant to the electrical grid.

In a similar vein, Fig. 9 shows how temperature varies over time. The temperature 25°C at the beginning of 0 s and stays that way until 1.5 s. Thereafter, the temperature abruptly rises to 30 °C, staying there at 3 s until the end simulations.

**Fig. 11:**
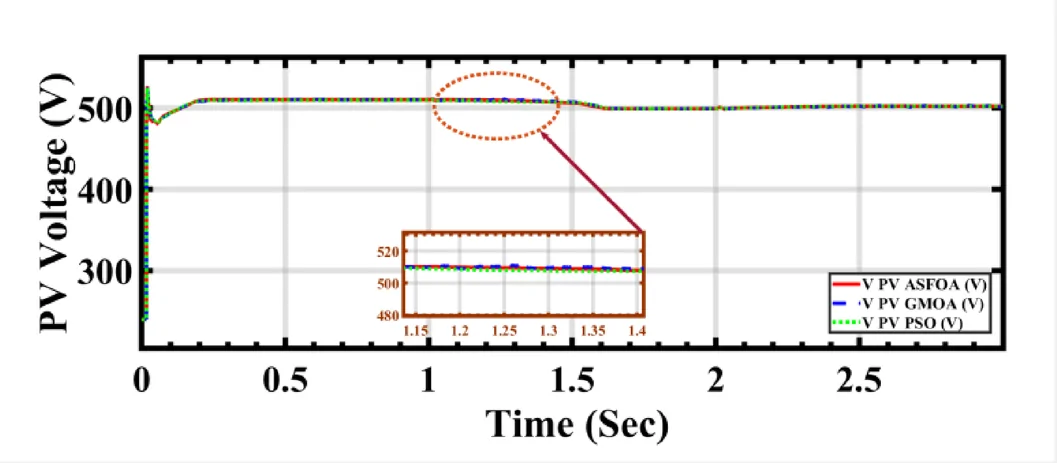
PV voltage Comparison between GMOA, ASFOA and PSO Optimization.

**Fig. 12:**
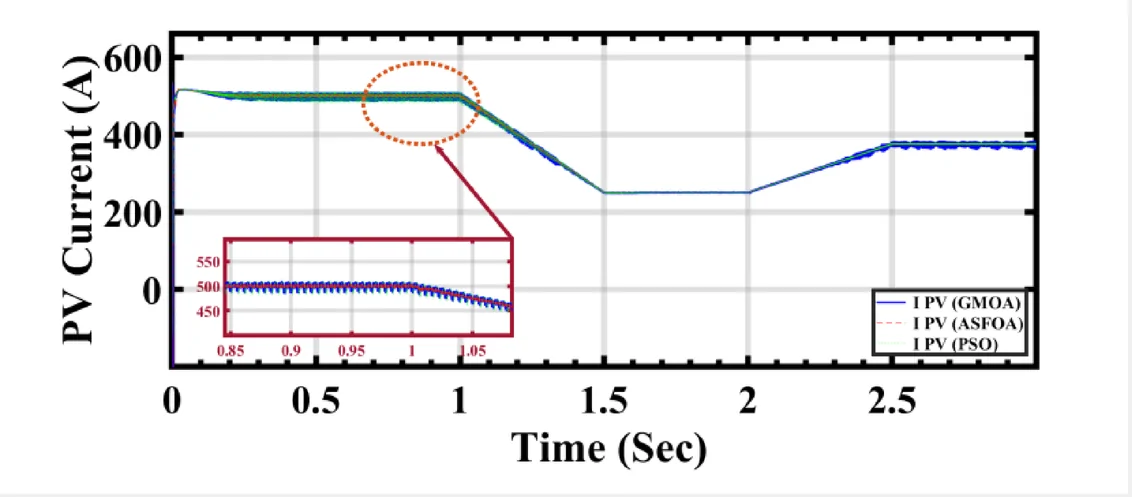
PV current Comparison between GMOA, ASFOA and PSO Optimization.

Figure 10 illustrate the step changes in WS are assumed in this case, which varies up and down as step profile with a mean value of 9 m/s with period of 3 s. This entire process involves collecting all wind energy and integrating it with the outputs of the solar cell. Ultimately, the grid relates to the total power production, thereby increasing the overall power supply and generation. Figure 11 illustrates the beginning of PV cells' voltage extraction through system control. Using both ASFOA and GOMA, Fig. 12, shows how the PV current performs during ramp differences in solar radiation.

**Fig. 13:**
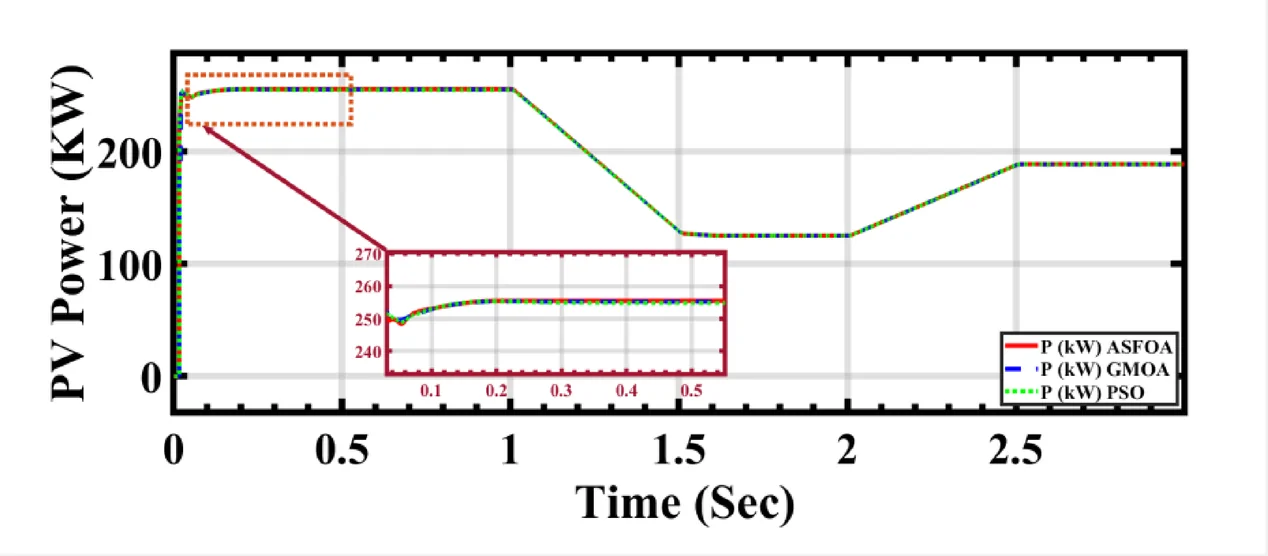
PV power Comparison between GMOA, ASFOA and PSO Optimization.

The PV power under the same radiation circumstances is displayed in Fig. 13, on the other hand, compared to ASFOA and PSO, ASFOA achieves more accurate and consistent maximum power extraction, especially during the transient phase and under changing operational conditions. Further demonstrates the controller’s ASFOA ability to accurately track the solar radiation profile, outperforming GMOA and PSO a in terms of response precision.

**Fig. 14:**
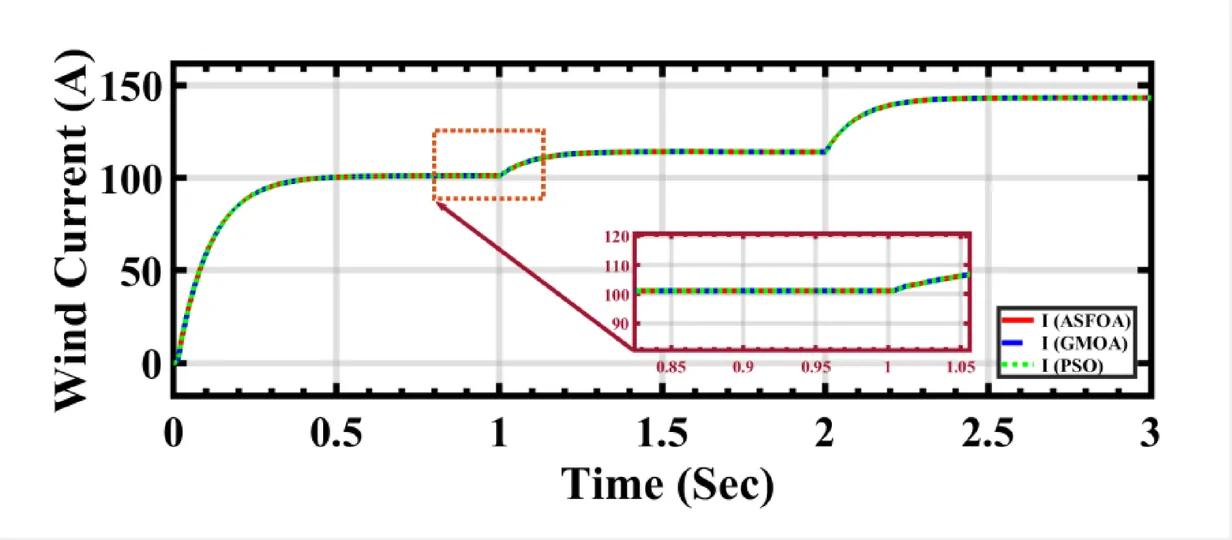
Production of wind current as the step wind profile changes.

Figure 14 indicates that the change in step wind profile clearly reveals changes in wind conditions, serving as a test of the adaptability and responsiveness of the GMOA, PSO and ASFOA Optimization algorithms. The wind current output produced by each algorithm is represented by the curves or lines on the graph when GMOA, PSO and ASFOA are compared side by side. A thorough assessment of how successfully both algorithms adjust to and reflect variations in the wind profile is made possible by this visual representation.

**Fig. 15:**
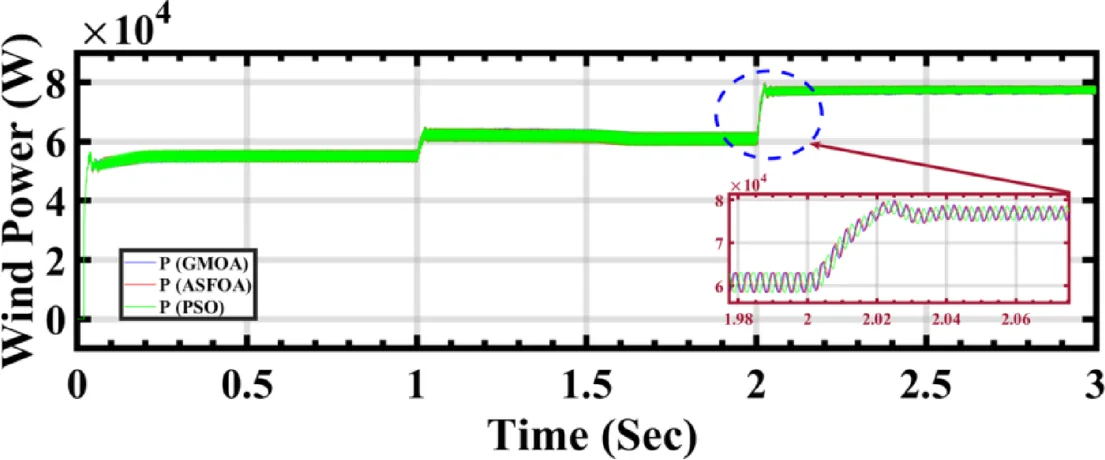
Wind Power Performance Comparison between GMOA, ASFOA and PSO Optimization.

The wind power outputs produced by each optimization technique are shown by distinct lines or curves on the plot in Fig. 15, which compares GMOA, PSO and ASFOA. A thorough examination of how well GMOA, PSO and ASFOA react to and reflect variations in the wind profile is made possible by this visual portrayal. The comparison seeks to show which approach captures wind energy more effectively, particularly when wind conditions fluctuate. The claims that ASFOA is more accurate than GMOA and PSO are supported by the numbers, which show how closely ASFOA matches the intended wind power outputs and highlights its greater precision and adaptability.

**Fig. 16:**
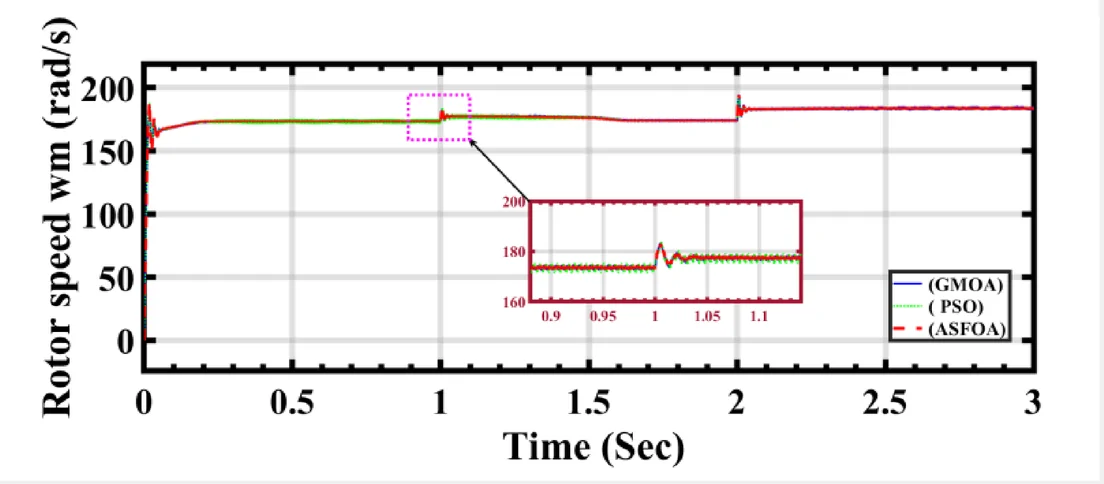
Wind rotor speed Performance Comparison between GMOA, ASFOA and PSO Optimization.

The various lines or curves on the plot in the comparison displayed in Fig. 16 indicate the rotor speeds produced by each optimization approach, GMOA, PSO and ASFOA. The comparison attempts to highlight the small differences in how each algorithm responds to and reflects changes in the wind profile to illustrate each algorithm's efficacy in maximizing rotor speed in a wind energy system.

**Fig. 17:**
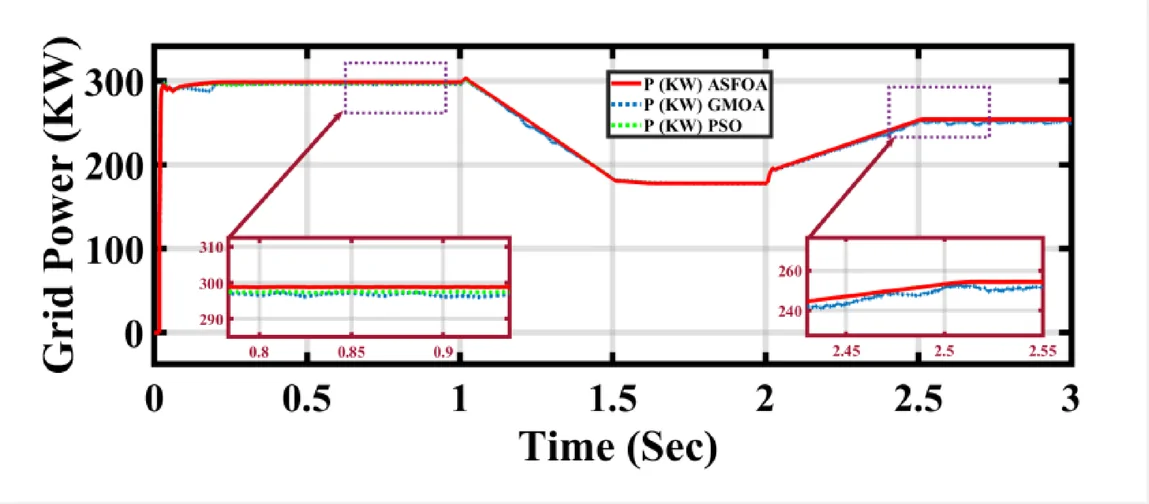
Grid Power Performance Comparison between GMOA, ASFOA and PSO Optimization.

Figure 17 illustrates how grid electricity behaves in relation to solar and wind energy under different weather situations. GMOA, PSO and ASFOA are three optimization methods that are compared. ASFOA performs better than GMOA and PSO in terms of accuracy. According to a thorough examination of the graph, ASFOA controls grid power more precisely and correctly than GMOA. In this application, accuracy refers to the optimization algorithm's capacity to provide a consistent and dependable supply of electricity to the grid, particularly while weather conditions are continually changing.

**Fig. 18:**
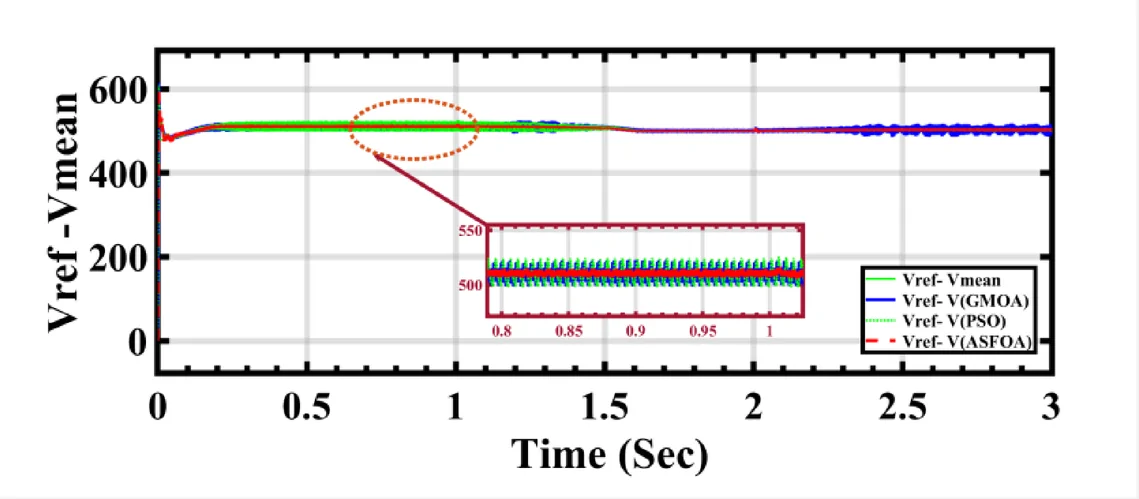
Comparison of DC-Link voltage regulation under GMOA, ASFOA and PSO optimizations.

Figure 18 displays the PV voltage produced by the wind turbine and PV array. To guarantee voltage stability, a DC link is attached at 500 V. There are differences between using ASFOA, GMOA and PSO, though. Both the voltage level and the waveform are disturbed by GMOA and PSO. ASFOA, on the other hand, connects to the DC link and successfully generates a constant voltage of 500 V.

**Fig. 19:**
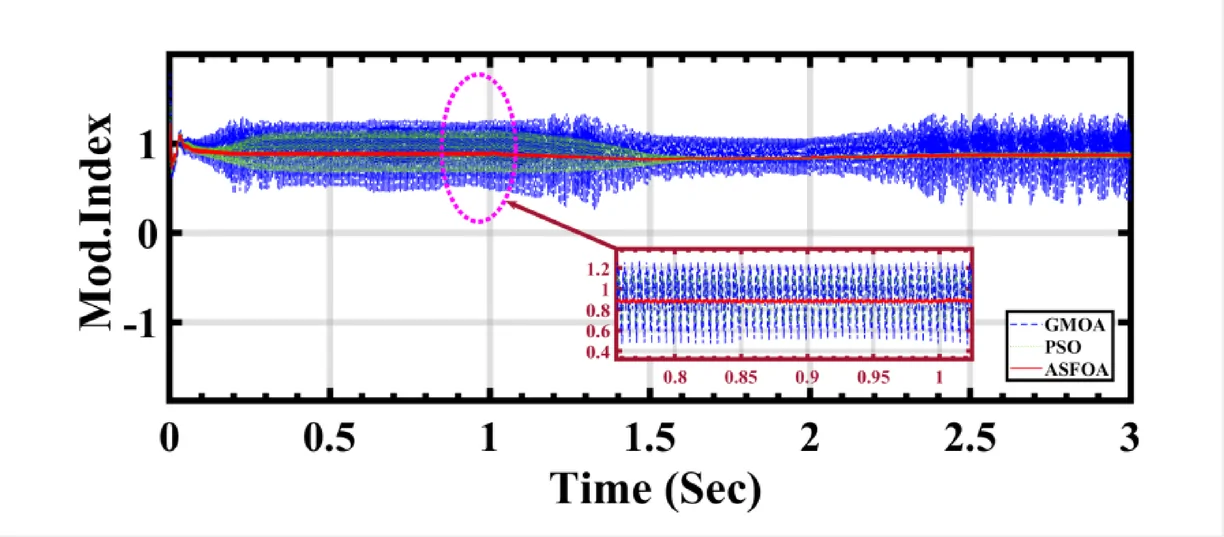
Modulation index dynamic response under GMOA, ASFOA and PSO –based PI controller tuning.

The performance of GMOA, ASFOA and PSO is compared in Fig. 19, which displays the modulation index control value for the AC/DC inverter. For the AC/DC inverter to effectively regulate the grid voltage, a PI controller is required. The input is provided with 500 V from the DC link to optimize the electricity generated by solar cells and wind turbines for a smooth connection to the electrical grid. The AC output voltage is increased from 260 V to 11 kilovolts for grid connection using a step-up transformer. It is crucial that this voltage stays constant throughout time and isn't adversely affected. The modulation index plays a crucial role in guaranteeing the smooth operation of the inverter and, consequently, the stability of the grid connection. These differences demonstrate how well ASFOA maximizes the modulation index and, as a result, the AC/DC inverter's capacity to maintain a steady grid voltage. The GMOA and PSO application's disruptions highlight potential challenges in achieving consistent and reliable voltage regulations under shifting circumstances.

**Fig. 20:**
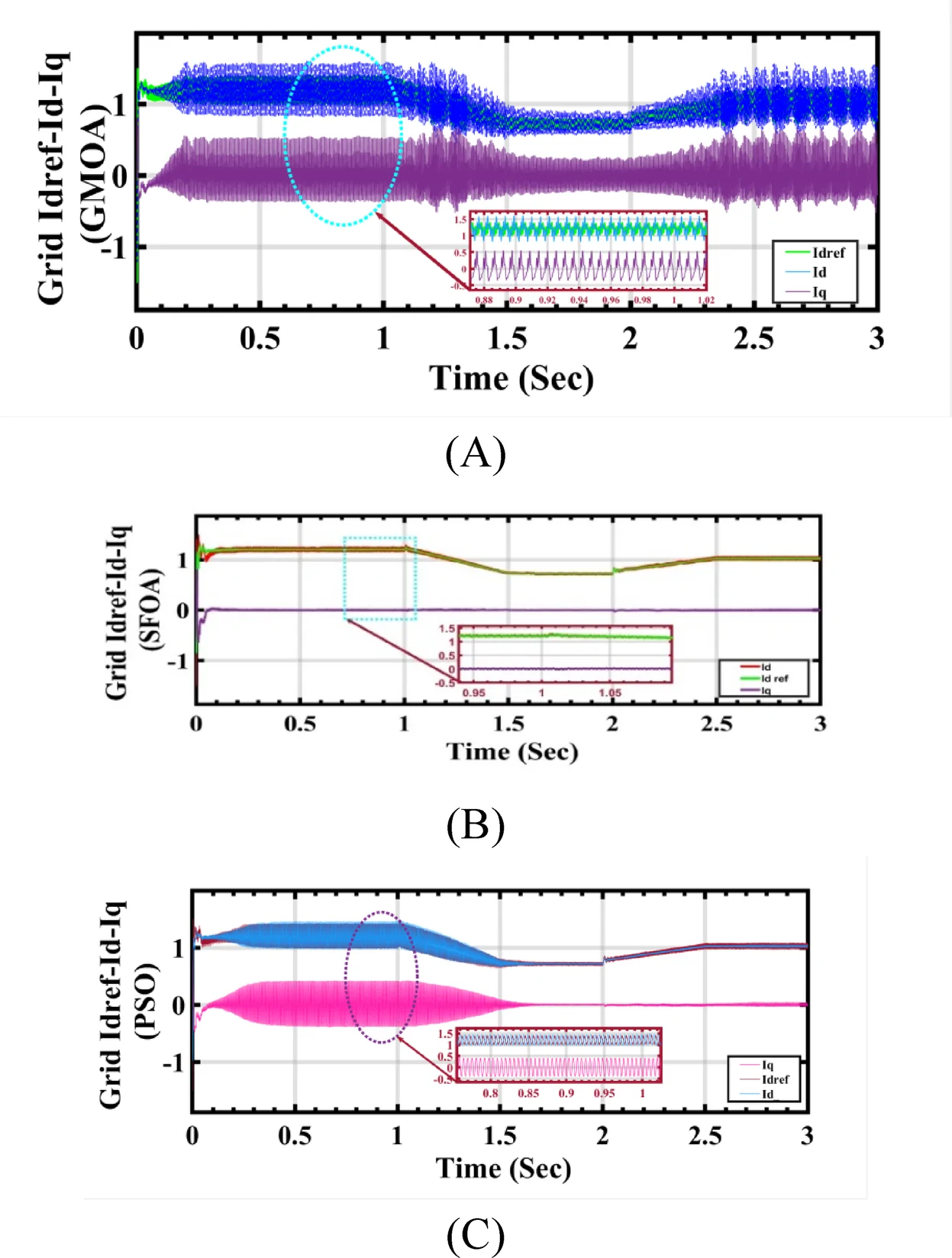
Comparison of d–q grid current tracking performance using (A) GMOA, (B) ASFOA and (C) PSO.

While maintaining ${I}_{q}$ (quadrature axis current) at zero, the graphs in Fig. 20, a, b and c illustrate how variations in solar radiation impact the values of ${I}_{d}$ (direct axis current) and ${I}_{dref}$(direct axis current reference). By relying only on one variable, ${I}_{d}$, this design decision streamlines the control process. Effective inverter control depends on the conversion process from the a, b, and c phases to the d and q axes, which produces the currents ${I}_{d}$ and ${I}_{q}$. Keeping ${I}_{q}$ at 0 makes controlling the inverter easier and more manageable. The current flowing along the quadrature axis is represented by Iq, whereas the current flowing along the direct axis is represented by ${I}_{d}$. There are discrepancies in the applications of GMOA, ASFOA and PSO, especially in the voltage output's stability and disturbance levels. When employing GMOA and PSO voltage disturbances become obvious, suggesting variations or deviations from the desired stability. On the other hand, by producing a stable voltage output, the use of ASFOA exhibits better performance.

**Fig. 21:**
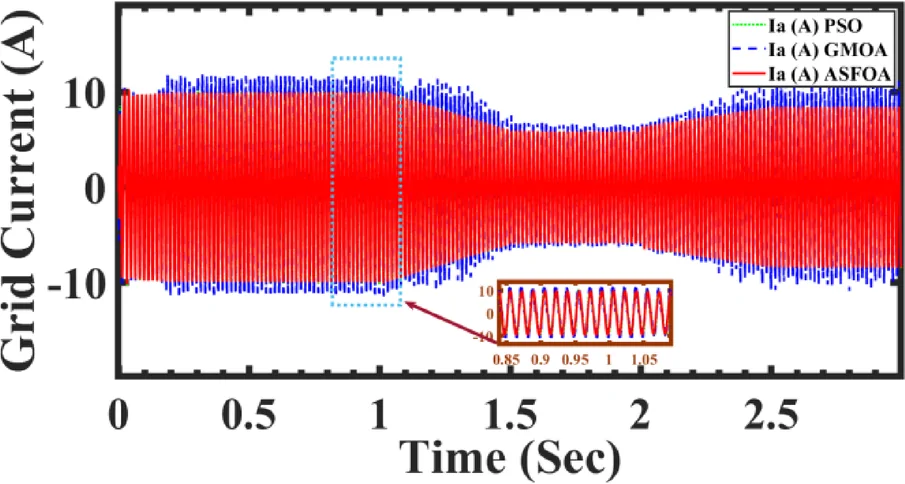
Grid phase current (Ia) waveform comparison under GMOA, ASFOA and PSO –based PI control tuning.

Figure 21 shows how grid current behaves under varying weather conditions related to solar and wind energy, with a focus on assessing the efficacy of three optimization algorithms: GMOA, ASFOA and PSO. The total reliability of the integrated renewable energy system is increased by the finding that ASFOA is more effective than GMOA and PSO in precisely and steadily controlling grid current.

**Fig. 22:**
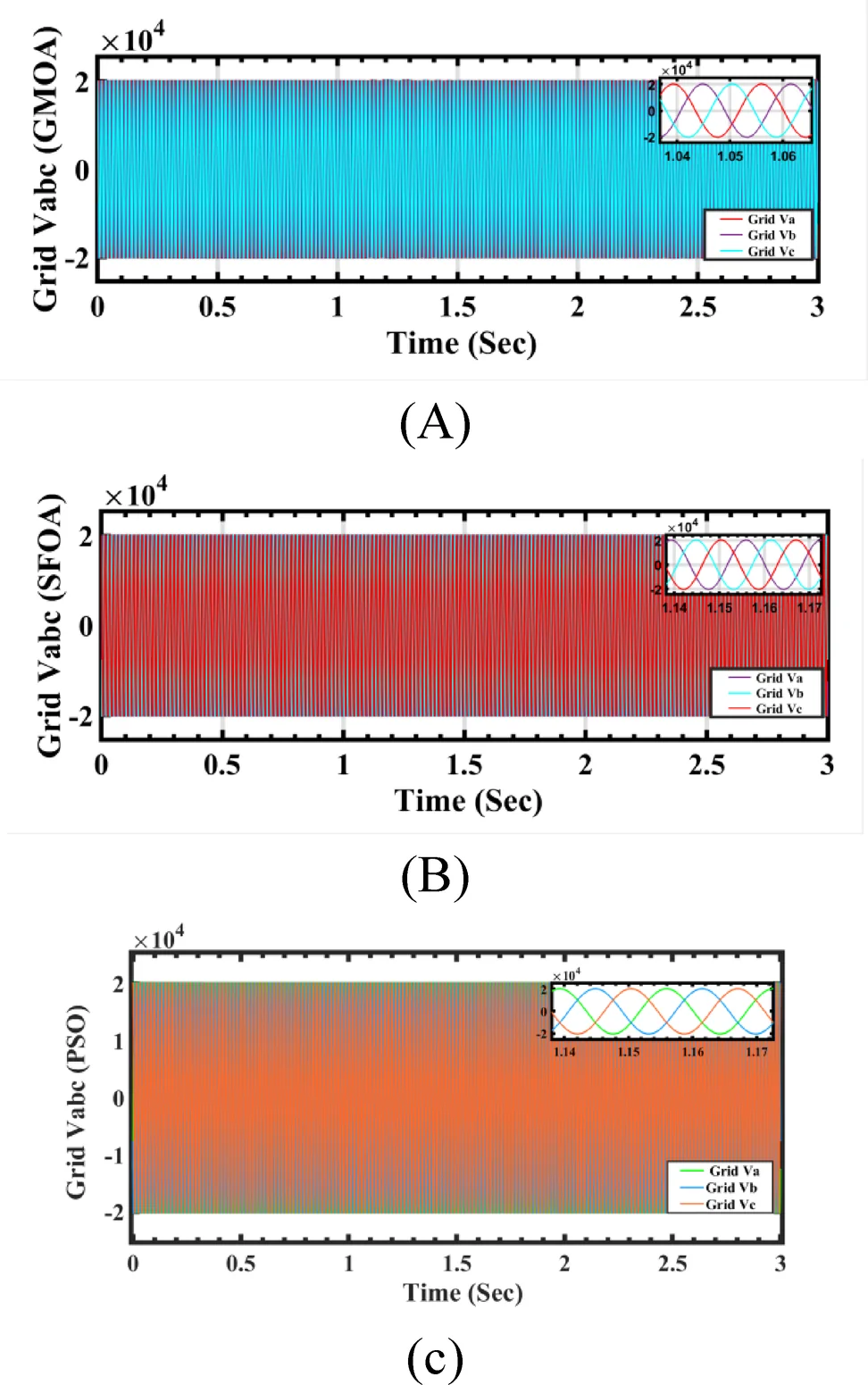
Three-phase grid voltage waveform (V_abc_) under PI controller tuning using: (**A**) GMOA, (**B**) ASFOA and (**C**) PSO.

With a focus on contrasting the employment of GMOA, ASFOA and PSO, Fig. 22a, b and c illustrates the grid voltage performance under varying weather conditions connected to solar and wind energy. ASFOA demonstrates grid voltage stability, it demonstrates the algorithm's ability to respond to shifting environmental conditions while ensuring a steady and reliable supply of electricity for the grid.

A comparison of the ASFOA and GMOA's performance under coordinated solar irradiance and WS profiles is shown in Table 3. The outcomes unequivocally show that ASFOA is better at optimizing grid-injected electricity under all operating circumstances. At high solar irradiance of 1000 W/m^2^ and a WS of 8.0 m/s, the ASFOA-based control strategy delivers a grid power of 298.72 kW, compared to 296.67 kW achieved by GMOA. This corresponds to a grid power improvement of approximately 3.06%, highlighting the faster convergence and more accurate tracking of the optimal operating point under steady-state conditions. When the irradiance level drops to 500 W/m^2^ with a WS of 8.5 m/s, the advantage of ASFOA becomes more pronounced. The grid power increases from 176.62 kW (GMOA) to 177.83 kW (ASFOA), yielding an improvement of about 3.69%. This improvement reflects the robustness of ASFOA in handling sudden irradiance reductions and maintaining effective power coordination between the PV and wind subsystems.

**Table 3:** Comparison of GMOA, PSO and ASFOA of PV, wind, and grid power.

Technique	Irradiance (W/m^2^)	WS (m/s)	Time (s)	P_PV_ (kW)	Wind power (kW)	Grid power (kW)
ASFOA	1000	8.0	0.80	241.6	57.0	298.72
GMOA	1000	8.0	0.80	241.6	55.0	296.67
PSO	1000	8.0	0.80	241.6	55.65	297.33
ASFOA	500	8.5	1.90	113.8	64.0	177.83
GMOA	500	8.5	1.90	114.2	62.5	176.62
PSO	500	8.5	1.90	113.9	63.03	176.93
ASFOA	750	9.5	2.60	191.0	75.0	266.0
GMOA	750	9.5	2.60	190.0	65.5	255.5
PSO	500	9.5	1.90	190.58	65.25	255.83

For the partial recovery stage at 750 W/m^2^ and a higher wind speed of 9.5 m/s, ASFOA achieves 266.0 kW of grid power, while GMOA reaches 255.5 kW. This represents the highest observed enhancement, with a grid power improvement of approximately 4.11%, indicating the superior adaptive behavior of ASFOA under combined deviations in solar radiation and WS. The results confirm that ASFOA consistently outperforms GMOA in terms of grid power injection, with improvement ratios ranging between 3 and 4.1%. These gains are attributed to the enhanced exploration–exploitation balance of ASFOA, which enables more effective coordination between PV generation and wind turbine dynamics, reduced power oscillations, and improved steady-state accuracy. Consequently, ASFOA proves to be a more reliable and efficient optimization technique for hybrid PV–wind grid-connected.

### Random profile for solar irradiance and wind speed profile

**Fig. 23:**
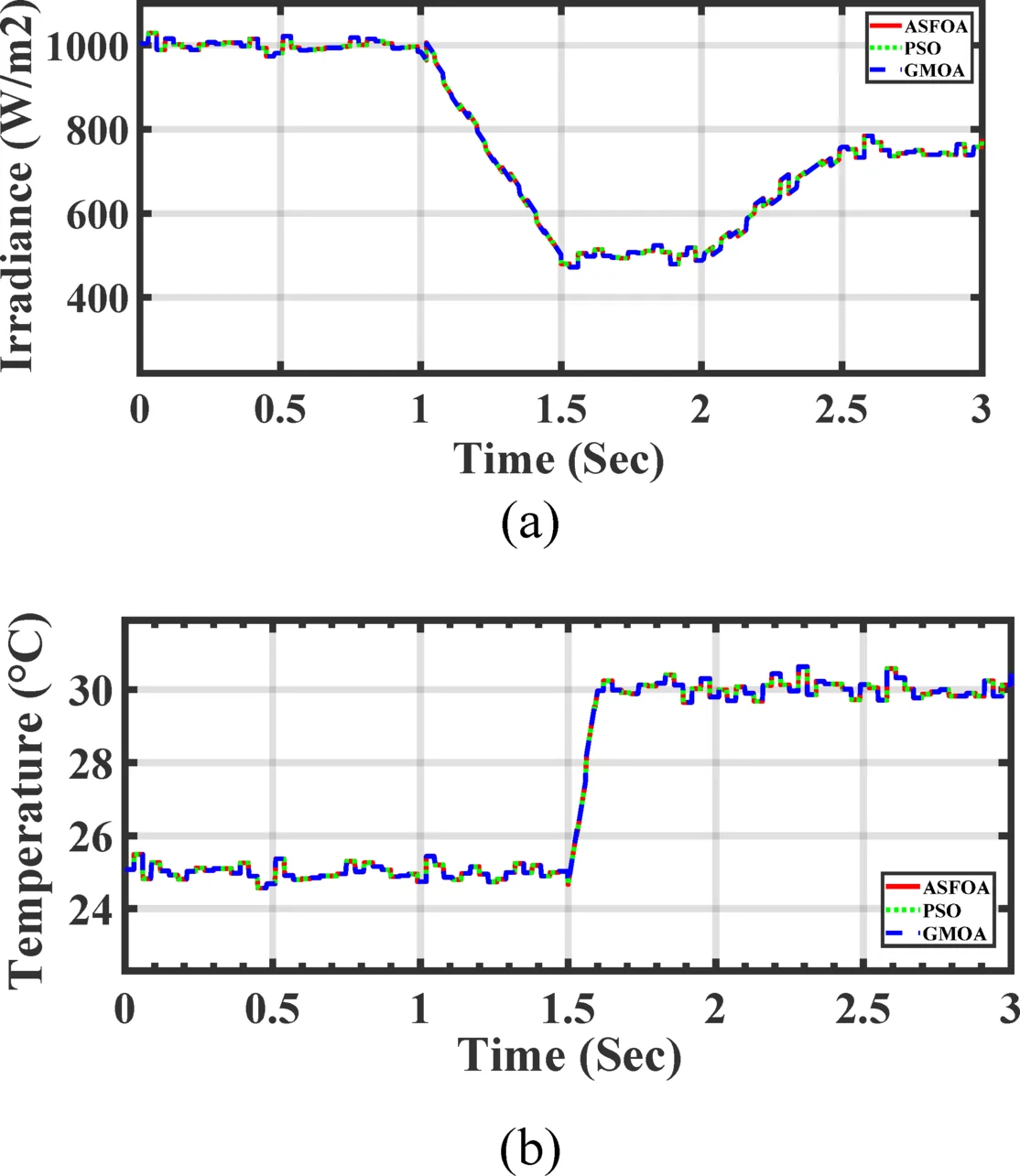
(**a**) Irradiance in a profile that is updated at random, (b) Temperature at random profile.

Figure 23a, b displays a sample profile with random variations solar energy around an average. Between 500 and 1000 W/m^2^. The ambient temperature in this profile changes from 25 to 30 degrees Celsius. Rainy days and cloud cover are two possible causes of these erratic variations in solar radiation. In these difficult circumstances, it is essential to address these unanticipated variations in radiation doses to guarantee the efficacy of the recommended MPPT approach and controller. The power voltage and current that the solar power plant outputs to the electrical grid are significantly impacted by variations in radiation.

**Fig. 24:**
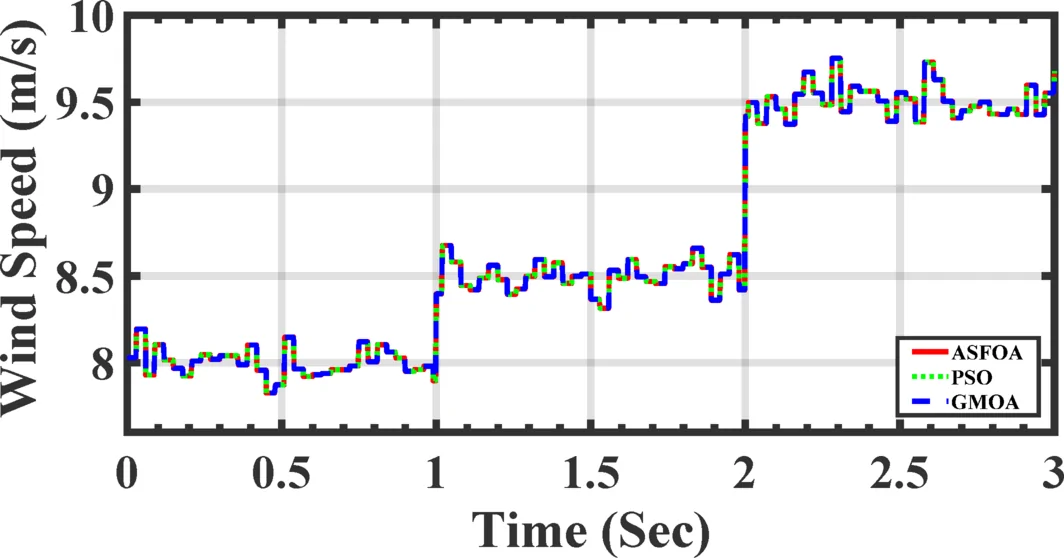
Random change in wind speed shape.

The feasibility of achieving MPPT under random variations in WS is explored, assuming a mean WS of 9 m/s and a turbulence intensity of 20%. The WS shape, which fluctuates randomly, is illustrated on Fig. 24.

**Fig. 25:**
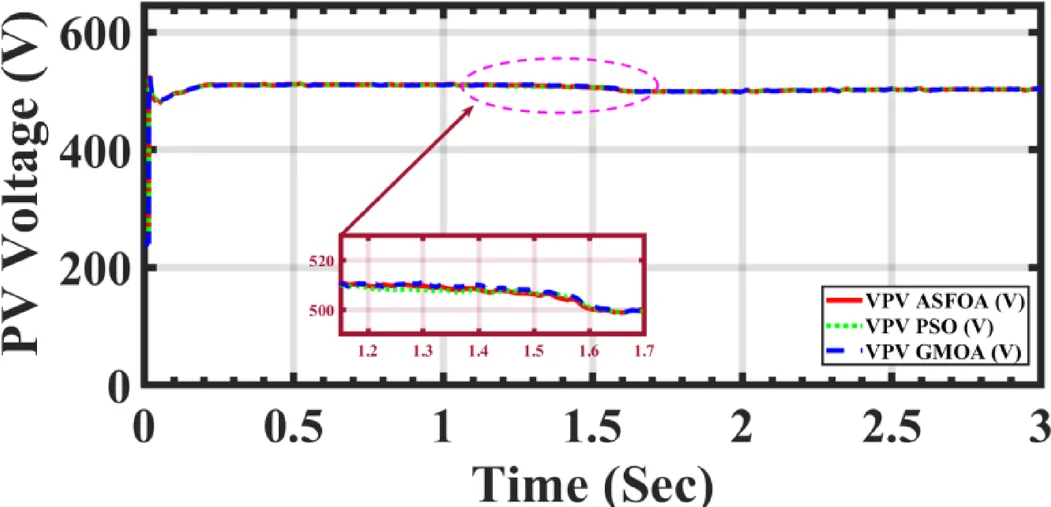
PV voltage performance during random variations in solar radiation.

The PV output voltage for each GMOA, PSO and ASFOA is displayed in Fig. 25. The graph shows that the ASFOA system outperforms the GMOA and PSO control method in terms of voltage output. The ASFOA system generates a voltage output that is more stable than the GMOA and PSO control system, especially at high temperatures. Furthermore, as the chart shows, the system responds more quickly under ASFOA than it does under GMOA and PSO control. As a result, ASFOA can be regarded as a superior control strategy for PV systems in terms of response time and voltage stability.

**Fig. 26:**
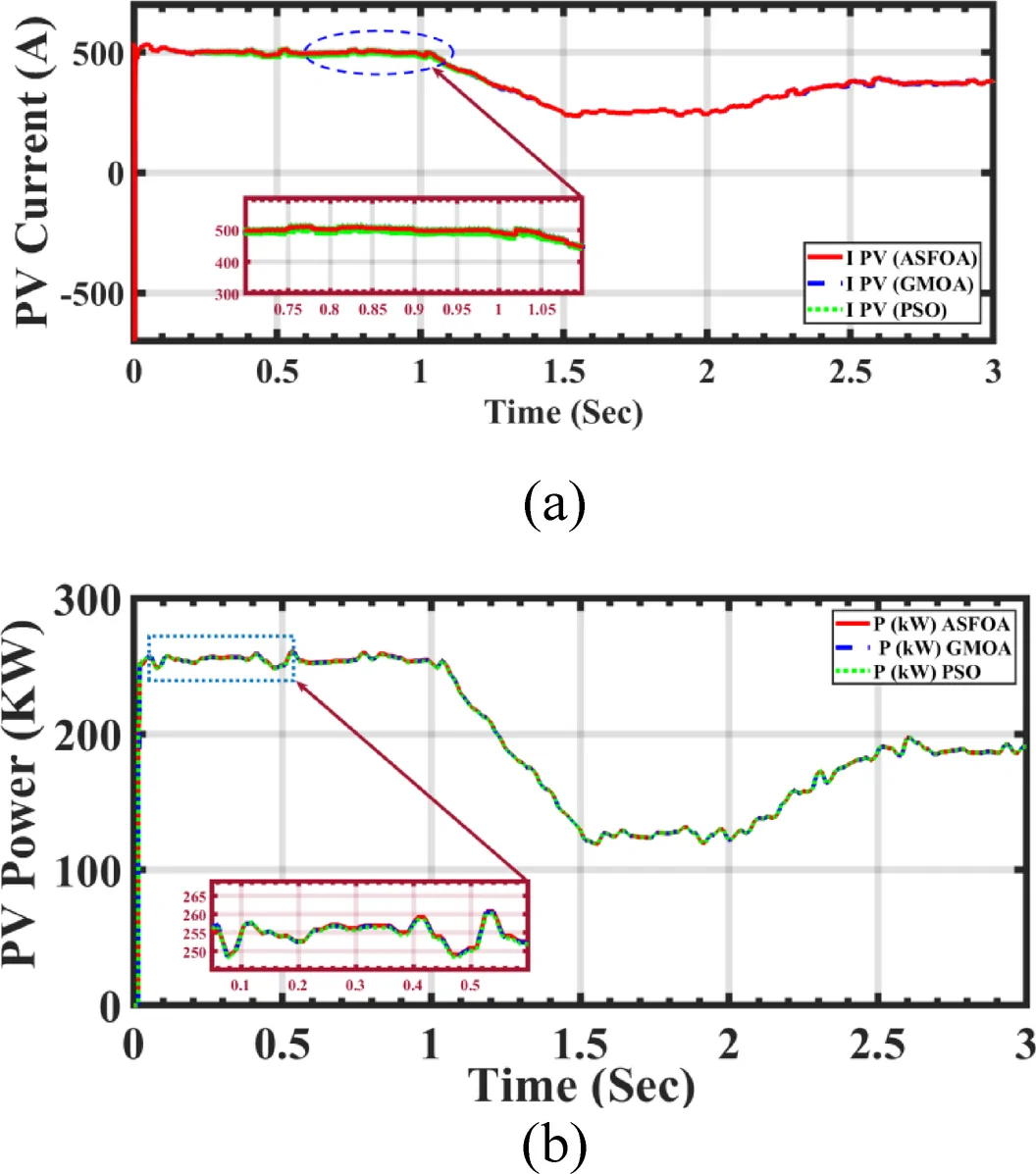
(**a**) PV current and (**b**) PV Power performance analysis with different proposed control techniques.

As illustrated in Fig. 26a. This enhanced tracking capacity guarantees the accurate and quick retrieval of the maximum PV current, which is necessary to optimize energy output in PV systems. Additionally, as Fig. 26b illustrates, the ASFOA methodology's PV output power outperforms the GMOA and PSO control strategy with fast random variations in solar radiation.

**Fig. 27:**
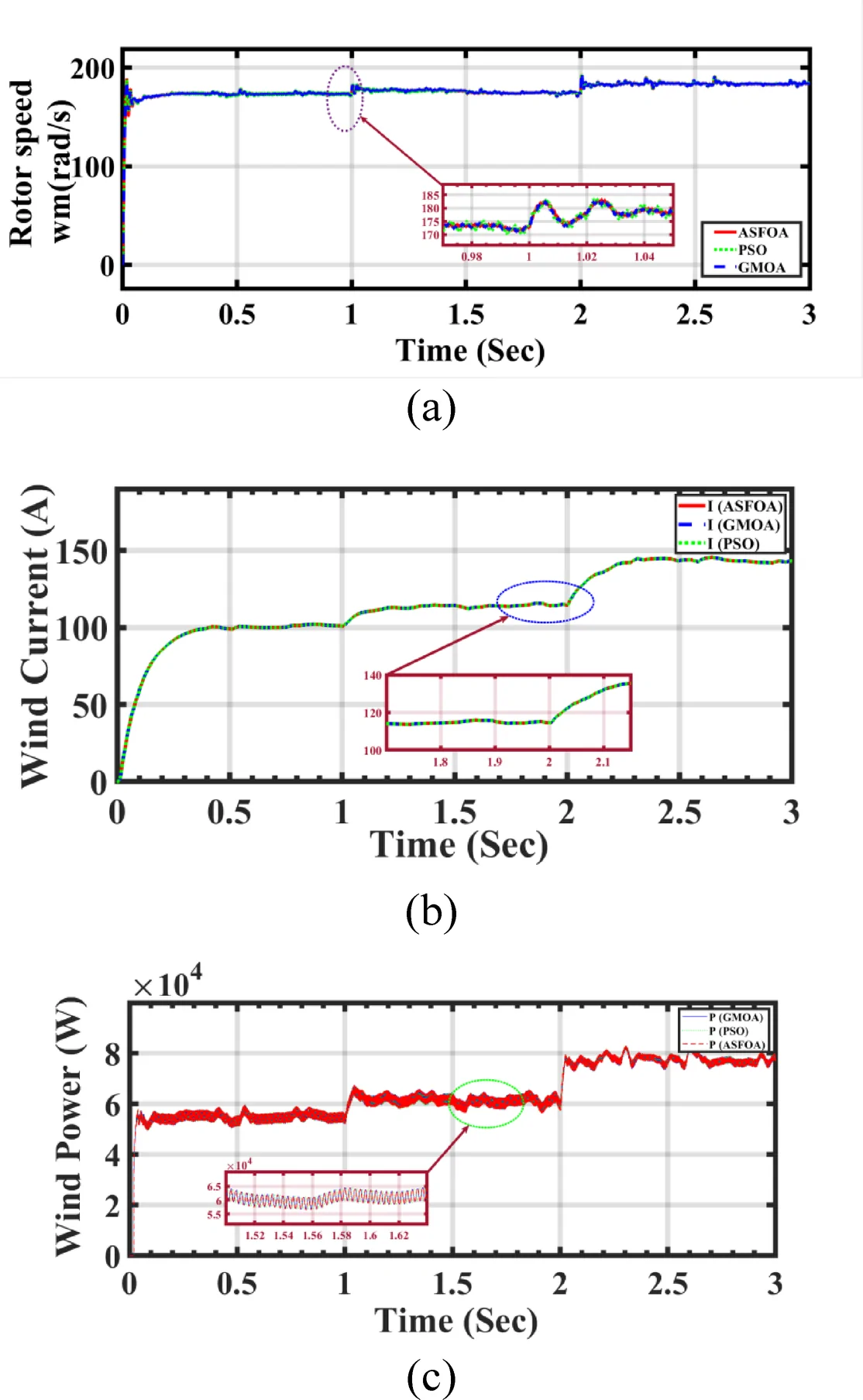
Response of wind turbine under random variation in WS shape.

The ability of the MSC to precisely follow the rotor speed and align it with the WS profile is shown in Fig. 27a, while Fig. 27b shows the wind current. Figure 27c displays the wind power that the MPPT was able to capture from the wind. According to the simulation results, the ASFOA system outperforms the GMOA in terms of tracking performance and oscillation rate. This enhancement can be ascribed to the ASFOA’s capacity to dynamically modify the controller parameters, guaranteeing more precise reaction to variations in WS and smoother operation.

**Fig. 28:**
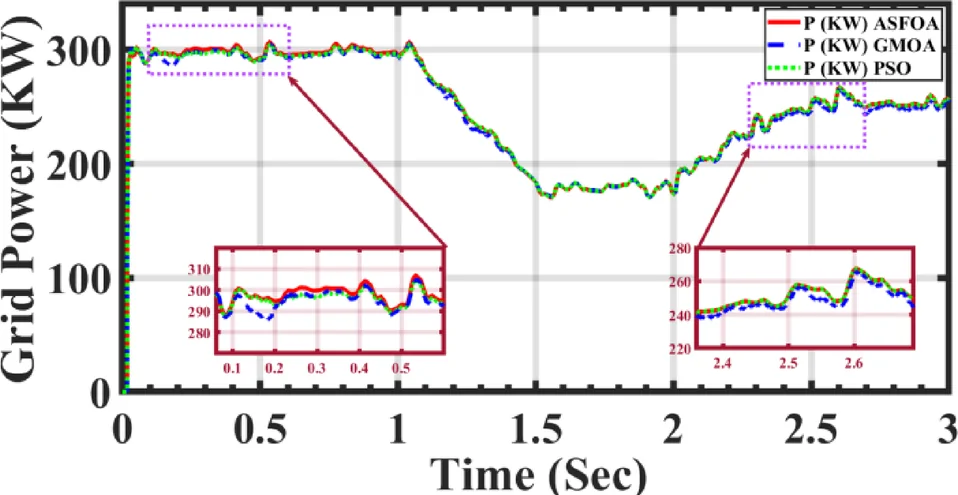
Grid power performance analysis with different proposed control techniques under random changing wind profile.

The graph in Fig. 28, shows how grid power behaves in random change irradiance, and weather situations related to Wind and Solar energy, with an emphasis on comparing the effectiveness of GMOA and ASFOA. It has been noted that ASFOA outperforms GMOA and PSO in terms of accuracy and stability in regulating grid power, hence increasing the overall dependability of the interconnected renewal every system's modulation index, as shown in Fig. 19, is almost equal to 1PU, demonstrating its continuous efficiency in stabilizing grid voltage efficiency and stability. The modulation index must be ≤ 1 to maintain stability.

According to comprehensive simulation results. Thus, in comparison to GMOA, PSO and ASFOA the ASFOA offers a lower oscillation rate and good tracking capacity at any conditions.

Table 4 illustrates the comparative performance of the ASFOA, GMOA and PSO optimization techniques under randomly varying solar irradiance and WS conditions, which represent a more realistic and challenging operating environment for hybrid PV–wind grid-connected. The results obtained consistently confirm the superior adaptability and robustness of the ASFOA-based control strategy.

**Table 4:** Comparison of GMOA, PSO and ASFOA under Random Solar Irradiance and WS Profile.

Technique	Irradiance (W/m^2^)	Wind Speed (m/s)	Time (s)	P_PV_ (kW)	Wind Power (kW)	Grid Power (kW)
ASFOA	980	8.0	1.20	241.0	71.5	312.5
GMOA	980	8.0	1.20	234.0	68.5	302.5
PSO	980	8.0	1.20	238.0	67.6	305.7
ASFOA	500	8.5	2.30	150.0	80.0	230.0
GMOA	500	8.5	2.30	144.0	76.5	220.5
PSO	500	8.5	2.30	147.0	78.6	225.6
ASFOA	750	9.5	3.00	193.0	75.5	268.5
GMOA	750	9.5	3.00	186.0	72.0	258.0
PSO	750	9.5	3.00	189.0	73.4	262.4

At an irradiance level of 980 W/m^2^ with a WS of 8.0 m/s, ASFOA injects 312.5 kW into the grid, whereas GMOA achieves 302.5 kW. This corresponds to an improvement of approximately 3.31%, demonstrating ASFOA’s enhanced ability to track the global optimum even under moderate random fluctuations in the input profiles.

Under reduced irradiance conditions of 500 W/m^2^ combined with a higher WS of 9.5 m/s, the advantage of ASFOA becomes more evident. The grid power increases from 220.5 kW with GMOA to 230.0 kW using ASFOA, yielding an improvement of about 4.31%. This improvement highlights the effectiveness of ASFOA in coordinating the nonlinear interaction between the PV array and wind turbine dynamics during abrupt environmental variations.

During the partial recovery phase at 750 W/m^2^ and 9.5 m/s, ASFOA continues to outperform GMOA by delivering 268.5 kW of grid power compared to 258.0 kW. Despite the unpredictable character of the solar irradiance and wind speed profiles, ASFOA maintains accurate and consistent power management, as evidenced by this improvement of roughly 4.07%.

## Proposed system of PV/wind hybrid system with real-time (OPAL-RT 4610)

**Fig. 29:**
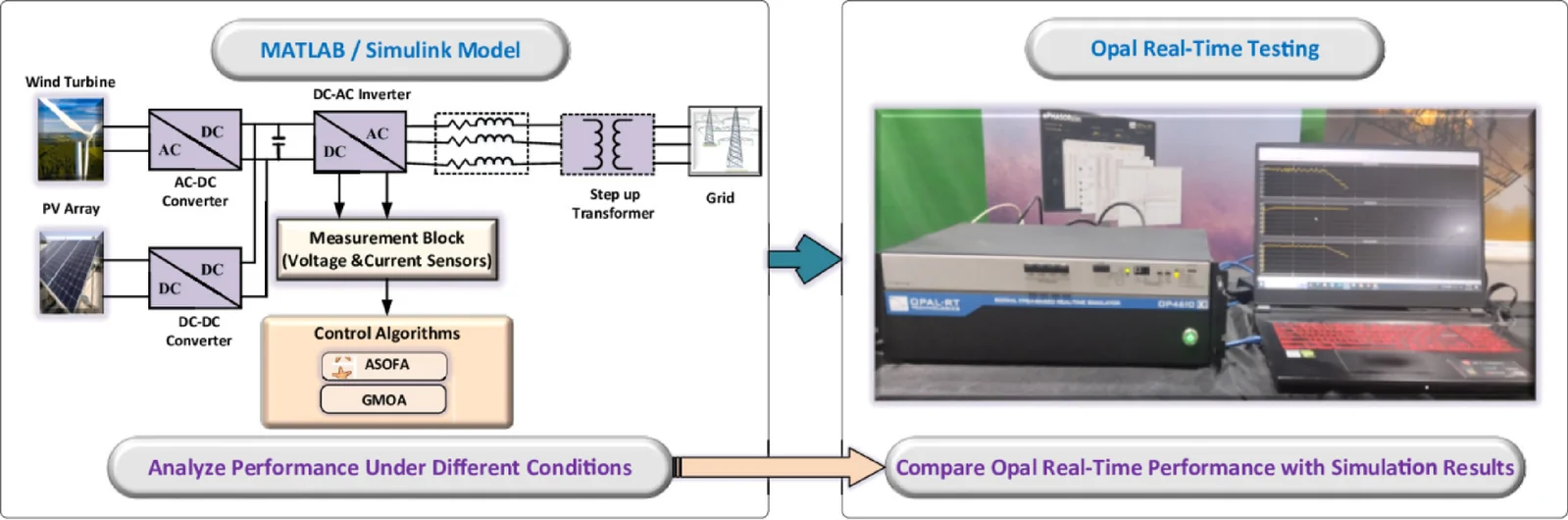
Hybrid PV-wind energy system developed & modeled using MATLAB Simulink and tested on OPAL-RT 4610 software under different operating scenarios.

The performance of a hybrid PV-wind energy system is analyzed using MATLAB/Simulink under different scenarios. Two optimization-based control methods, ASOFA, GMOA and PSO are utilized to get more power from both renewable sources and to make the system easier to regulate. To further confirm that the proposed control methods work, the produced model is being used on the OPAL-RT real-time simulation platform. This makes it possible to perform an evaluation of the behavior of the system in real time, as shown in Fig. 29. The system's performance is tested in different environmental conditions, such as changes in wind speed, temperature, and solar irradiation. To make the weather changes more realistic, these changes are used in two ways: step profiles and random profiles. The results from the MATLAB simulations are then compared to the real-time data from OPAL-RT. This shows that the proposed control strategies are legitimate and that the optimization approaches used are better.

The proposed control system was first developed and verified in the MATLAB/Simulink environment before being deployed to the OPAL-RT real-time simulator. After the model compilation process, the control algorithms and power system model were executed in real time using a fixed simulation step to ensure deterministic execution. The optimized PI controller parameters obtained using ASFOA, GMOA and PSO were directly implemented within the real-time model, allowing the controller to interact continuously with the plant model under different operating conditions. This implementation enables accurate assessment of the controller's dynamic performance, including DC-link voltage regulation, grid synchronization, active and reactive power control, and maximum power extraction, while preserving real-time computational performance and execution stability.

The OPAL-RT platform provides a deterministic real-time execution environment in which the power system model and control algorithms operate synchronously at each sampling interval. This execution framework minimizes computational delay and guarantees that the optimized controller gains are evaluated under realistic operating conditions. Consequently, the obtained real-time results closely represent the expected practical performance of the proposed control strategy and provide additional confidence in its applicability for grid-connected renewable energy systems.

The OPAL-RT 4610 real-time simulation platform was used to confirm the real-time performance of the proposed controllers. This hardware-based environment enables the evaluation of control algorithms under real-world conditions that are not fully represented in offline simulations, such as constrained sampling intervals, communication delays, and numerical accuracy impacts. This type of evaluation ensures that the recommended ASFOA, GMOA and PSO optimized controller is theoretically efficient and practically reliable for real-world power system applications.as shown by the OPAL-RT experimental setup in Fig. 29. It should be mentioned that the OPAL-RT studies' findings are susceptible to intrinsic latency and error components that are not present in conventional offline implementations. The real-time simulator OPAL-RT serves as the basis for these answers. As seen in Fig. 29, the results from the MATLAB simulation program and the OPAL-RT real-time simulator exhibit a notable degree of similarity.

Again, it should be mentioned that the OPAL-RT hardware experiences minor delays and dead-time effects due to real-time signal processing and data transfer. Despite these genuine nonlinearities, the OPAL-RT platform's practical findings closely matched the MATLAB/Simulink simulations. Therefore, it is believed that the proposed model and controller architecture are accurate and dependable enough for real-world operating conditions. About the real-time validation and HIL/OPAL-RT configuration. As requested, we have revised the manuscript to include quantifiable and unambiguous evidence.

The OPAL-RT 4610 real-time simulator was used to run the hybrid PV–wind system model, which included PV system, turbine aerodynamics, generator dynamics, and power electronics. Depending on the HIL situation, either the simulation host or the actual controller was used to implement the MPPT controller ASFOA, GMOA and PSO optimized controller. For every real-time run, the Ode4 solver with a fixed step of 10 µs was utilized. The OPAL-RT CPU load stayed at about 75% during peak simulation, providing enough leeway for real-time determinism. With an AMD RyzenTM CPU with six cores running at 3.8 GHz and a Xilinx® Kintex®-7 FPGA, the OP4610XG features a powerful computational architecture. Throughout the experiments, no overruns were found. The RT-LAB diagnostic panel was used to keep an eye on overrun counters. Repeated runs that produced the same timing and signal output confirmed that the simulation was entirely deterministic under the given time step. The HIL configuration had ADC/encoder inputs for measured current, voltage, and rotor speed signals as well as PWM outputs from OPAL-RT driving the hardware controller. The diagram of I/O topology Fig. 29 shows the I/O topology diagram. to provide a clear example of signal routing between external hardware and OPAL-RT. Four fiber-optic SFP sockets, up to 128 high-speed digital and analog I/O lines, and a variety of communication protocols are all supported. In addition to OPAL-RT, only the ASFOA, GMOA and PSO optimized controller MPPT algorithm was run in real-time on the host.

**Fig. 30:**
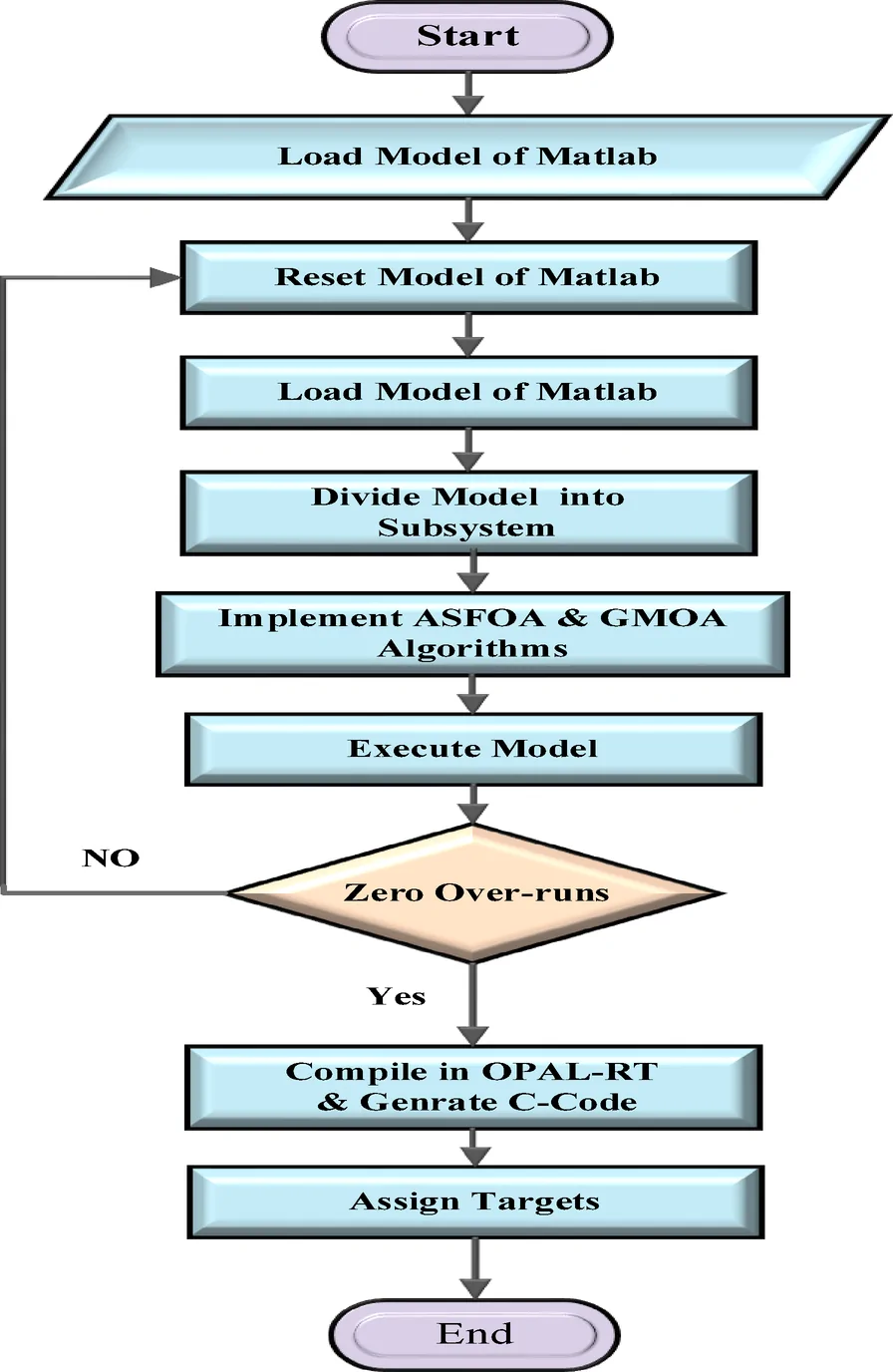
OPAL-RT Implementation Procedure.

The flowchart shown in Fig. 30 illustrates the main actions followed to implement the proposed hybrid PV–wind system using OPAL-RT real-time platform. The process starts with loading and resetting the MATLAB model to ensure proper initialization. After that, the optimization algorithms, namely ASFOA, GMOA and PSO, are integrated into the model to enhance the control performance and improve maximum power extraction from the renewable sources.

### Ramp profile for solar irradiance and step profile for wind speed

**Fig. 31:**
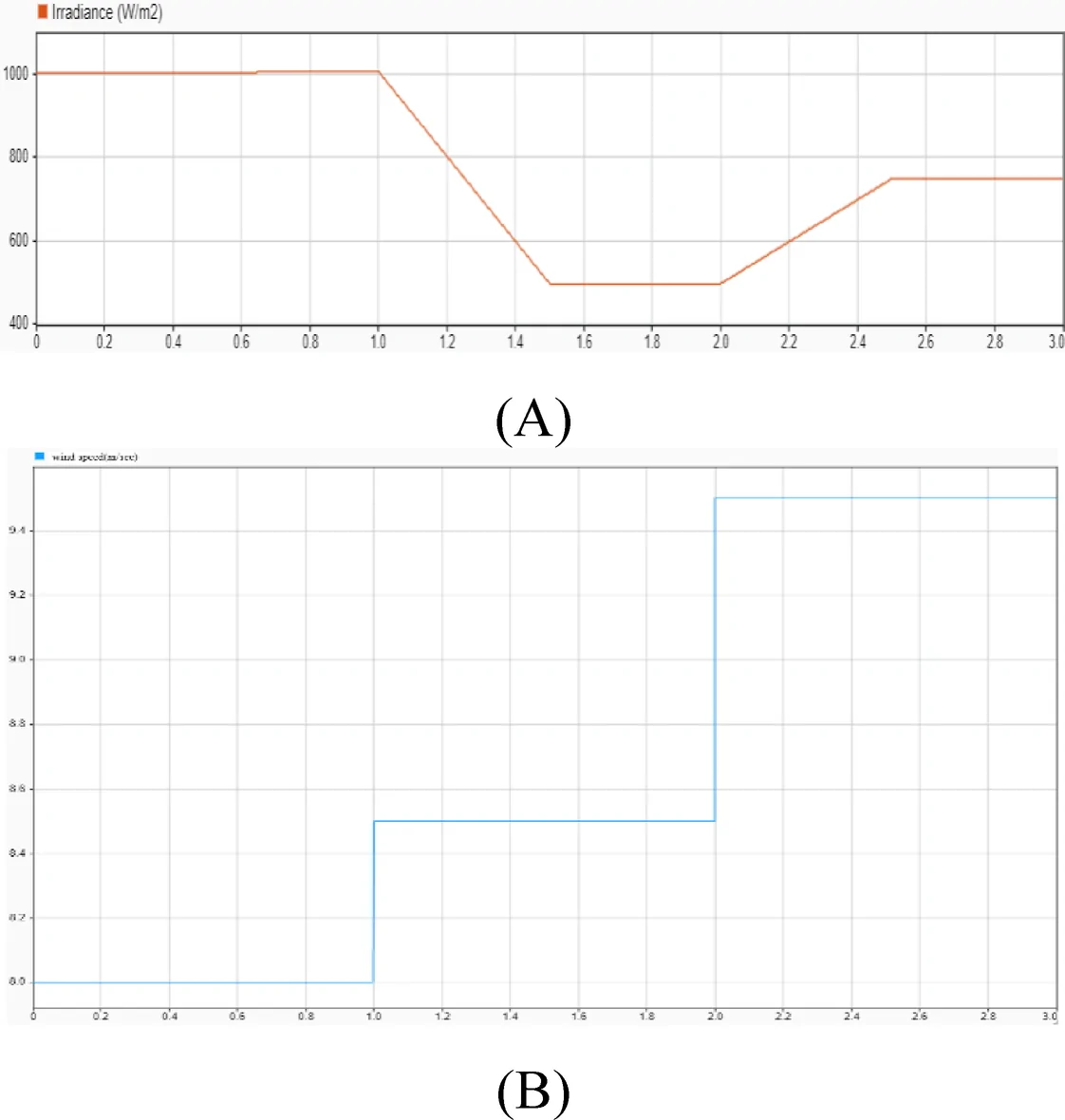
(**A**) Variations in PV radiation and temperature, (**B**) Changes in wind speed, in (OPAL-RT real time simulator).

**Fig. 32:**
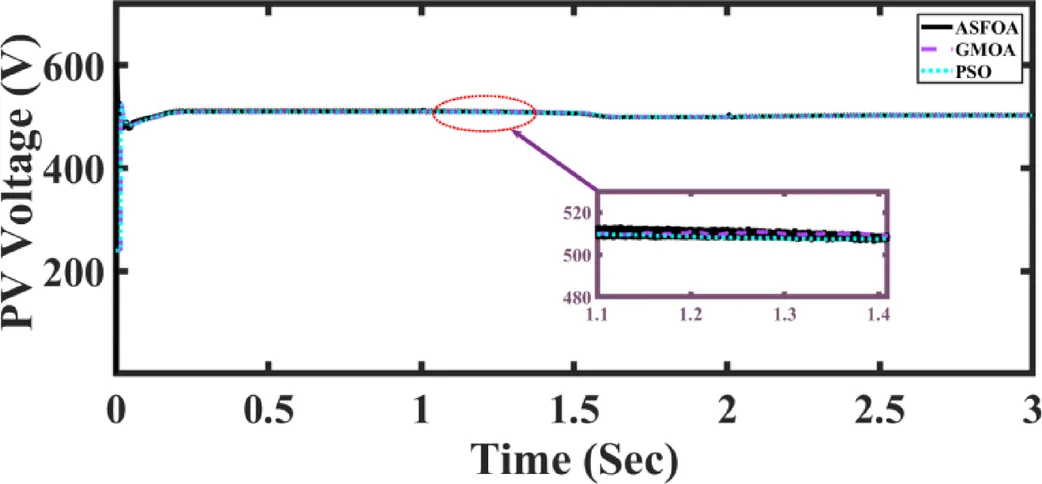
PV voltage comparison between GMOA, ASFOA and PSO optimization techniques using OPAL-RT real-time simulator.

**Fig. 33:**
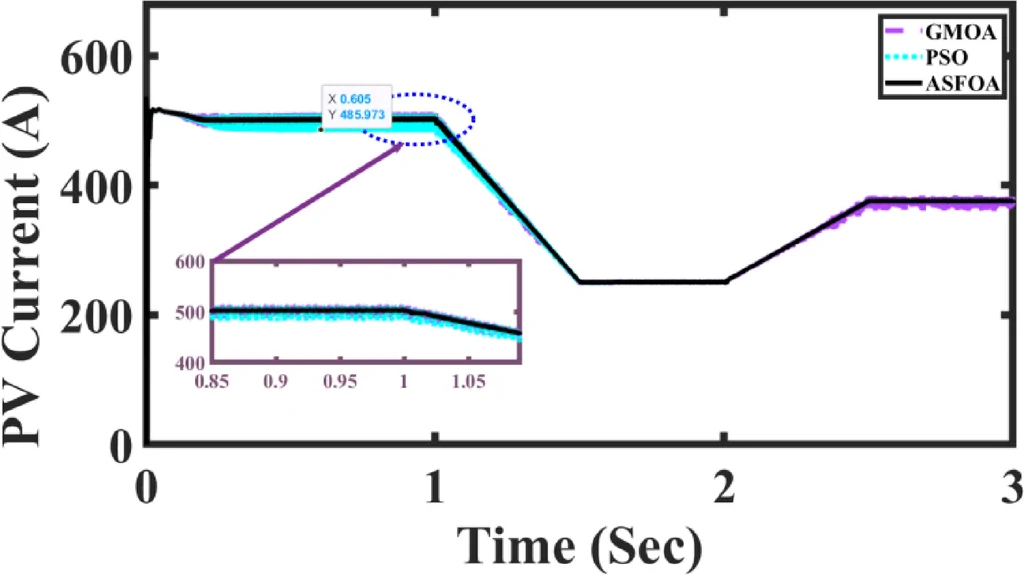
PV current response under GMOA, ASFOA and PSO optimization techniques simulated using OPAL-RT.

The hybrid PV-wind system was simulated in real time using the OPAL-RT real-time simulator in order to assess its dynamic presentation under realistic environmental variations. In addition to a ramp-varying profile for solar irradiation, step-change profiles for wind speed were employed, as illustrated in Fig. 31. The PV output voltage obtained with the GMOA, PSO and ASFOA control strategies applied on the real-time simulation platform OPAL-RT is shown in Fig. 32. The results clearly demonstrate that the ASFOA-controlled system outperforms the GMOA and PSO -based control method in terms of voltage output characteristics. Specifically, voltage produced under ASFOA control is more stable, especially at high temperatures. In addition, when compared to the GMOA and PSO -controlled system, the ASFOA-controlled system responds more quickly to environmental changes. So, ASFOA is a better way to control PV systems because it makes the voltage more stable and the response time faster when the weather changes in real time. Figure 33 shows how the PV current changes when the amount of solar irradiance changes. This was done using both the ASFOA, GMOA and PSO control strategies on the OPAL-RT real-time simulator. Both control approaches show that PV current follows solar irradiation. However, the ASFOA-based controller responds faster and smoother than GMOA and PSO. The current obtained with SFOA has smaller oscillations and higher stability during irradiance transitions. The real-time implementation on OPAL-RT confirms the ASFOA control strategy's environmental disparity tracking accuracy and reliability.

**Fig. 34:**
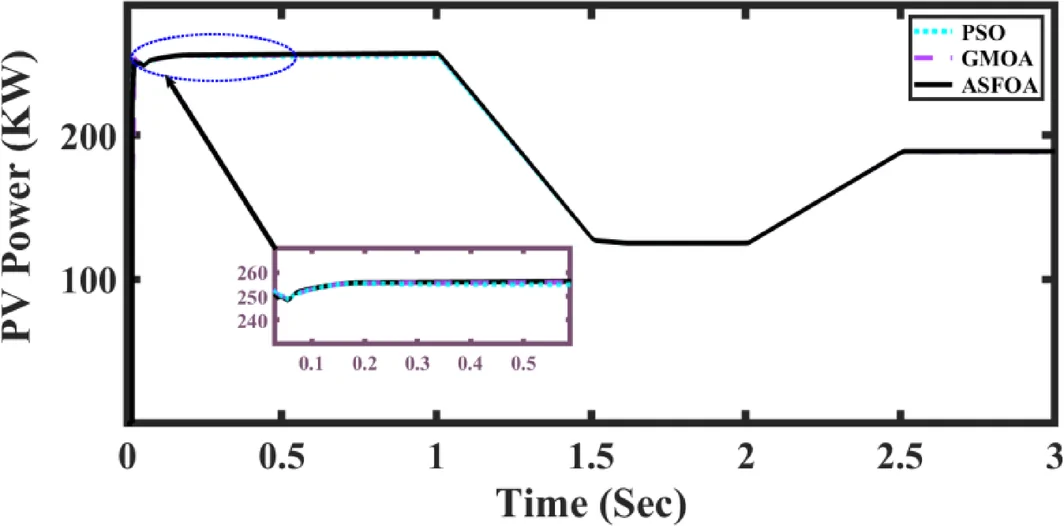
PV power response under ramp variations in solar radiation using GMOA, ASFOA and PSO, simulated in real-time with OPAL-RT 4610.

Figure 34 illustrates the PV output power under identical solar irradiance conditions utilizing both the ASFOA, GMOA and PSO management techniques, implemented on the OPAL-RT platform. The results demonstrate that the ASFOA controller achieved better accuracy and consistency in maximum power extraction, especially during transient periods and under fluctuating operating conditions. The study's results indicate that the ASFOA controller can accurately track changes in solar irradiance. Moreover, the real-time of OPAL-RT supports the effectiveness and reliability of the proposed ASFOA control method.

**Fig. 35:**
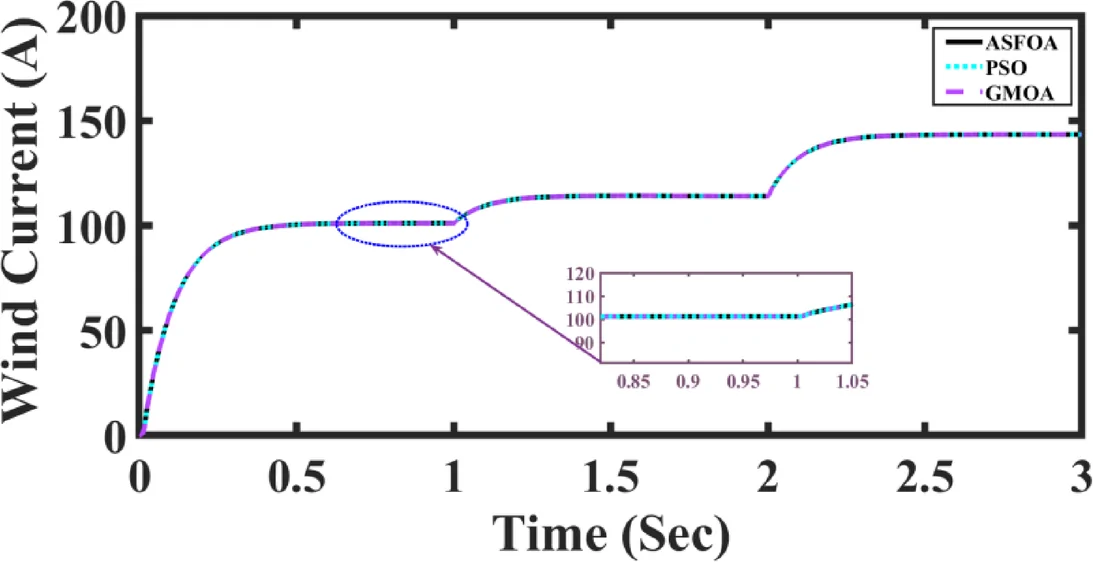
Wind current as the step wind profile changes.

The step-change wind speed profile that was utilized to examine the dynamical response and flexibility of the optimization methods is graphically represented in Fig. 35. It is possible to make a direct comparison of the tracking performance of GMOA, PSO and ASFOA under different wind conditions according to the illustrations of the matching wind current responses that were acquired using both methods. The ASFOA algorithm can offer a wind current response that more closely reflects the expected values, particularly during transient periods in which variations in wind speed occur. A higher tracking accuracy and quicker adaptation time are both indicated by this. In addition, the system was tested in real-time using the OPAL-RT tool to show that the proposed method works. The simulation results and the experimental results from the OPAL-RT setup agree very well, which proves that the ASFOA-based control approach is reliable and strong.

**Fig. 36:**
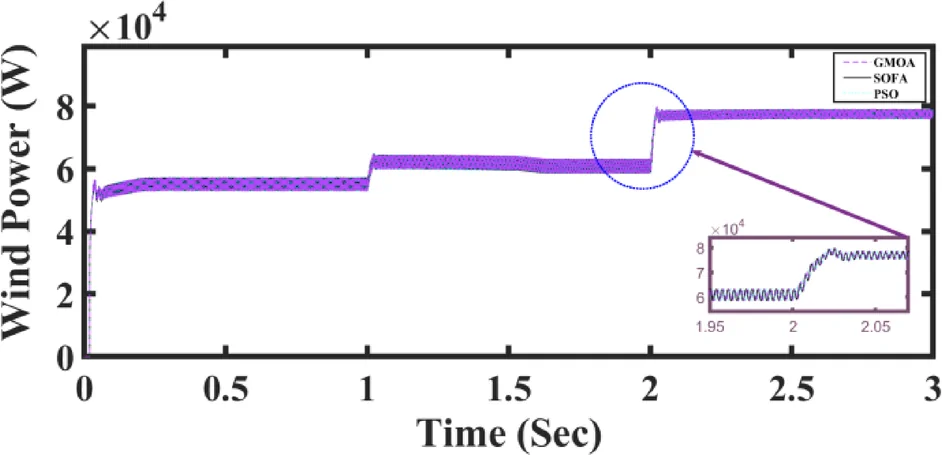
Wind Power Performance Comparison between GMOA, ASFOA and PSO Optimization under OPAL-RT Simulator.

In Fig. 36, we observe the results of the GMOA, PSO and ASFOA optimization algorithms generating wind power. The curves show the power response of each approach under different wind conditions, allowing for a direct comparison of their tracking capabilities. The ASFOA algorithm outperforms the GMOA and PSO method in terms of accuracy and adaptability, as it produces wind power outputs that are more in line with the predicted values. The proposed method was also validated in a real-time environment utilizing the OPAL-RT platform, which further demonstrated the efficacy of the ASFOA-based optimization approach. There was a strong agreement between the experimental findings and the simulation results.

**Fig. 37:**
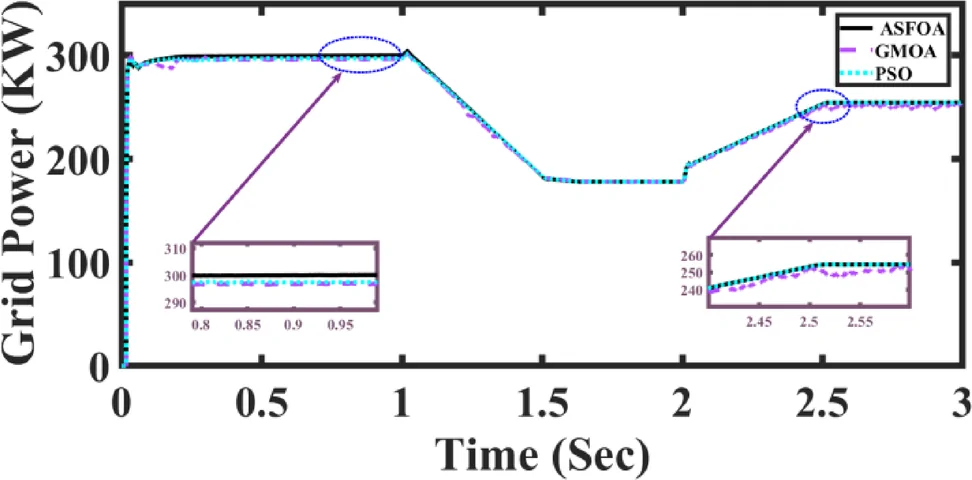
Grid Power Performance Comparison between GMOA, ASFOA and PSO Optimization (OPAL-RT 4610).

**Fig. 38:**
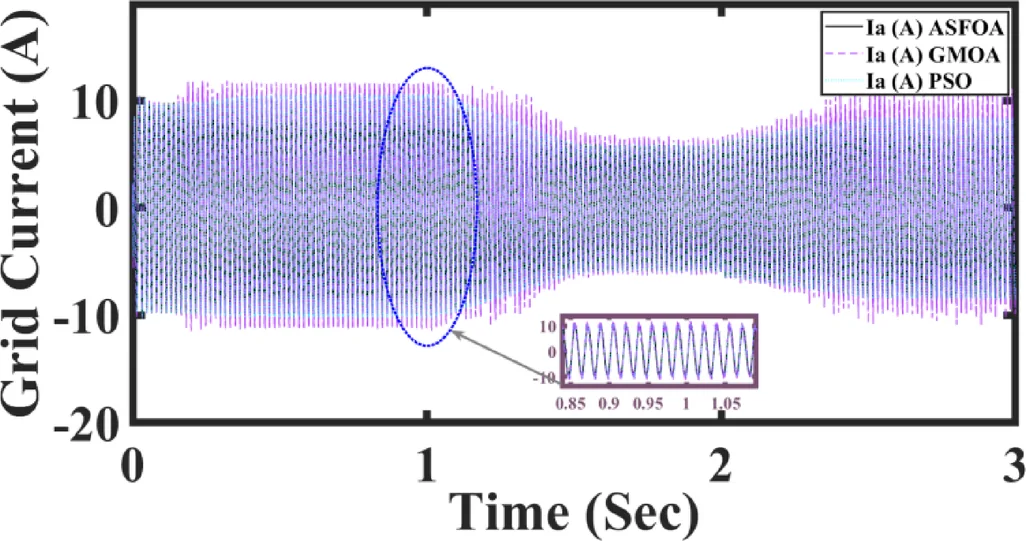
Grid phase current (Ia) waveform comparison under GMOA, ASFOA and PSO using (OPAL-RT 4610).

The behavior of the grid power in respect to the contributions of solar and wind energy is depicted in Fig. 37. This behavior is shown in relation to the different weather conditions. To determining how effective the GMOA, PSO and ASFOA optimization strategies are in terms of controlling the amount of power that is delivered to the grid, a comparison between the two is offered. In contrast to the GMOA and PSO techniques, the ASFOA method exhibits superior performance, particularly concerning accuracy and stability. The provided figure illustrates ASFOA's capacity to offer more precise control over grid power, thereby facilitating smoother power regulation and enhanced adaptability to dynamic environmental fluctuations. Specifically, this precision implies that the optimization method can maintain a stable and reliable grid power supply, even amidst variations in temperature, wind speed, and solar irradiance. Furthermore, the simulation outcomes were validated through real-time implementation on the OPAL RT platform, which confirmed the effectiveness and reliability of the proposed ASFOA-based control method under practical operating conditions (Fig. 38).

**Fig. 39:**
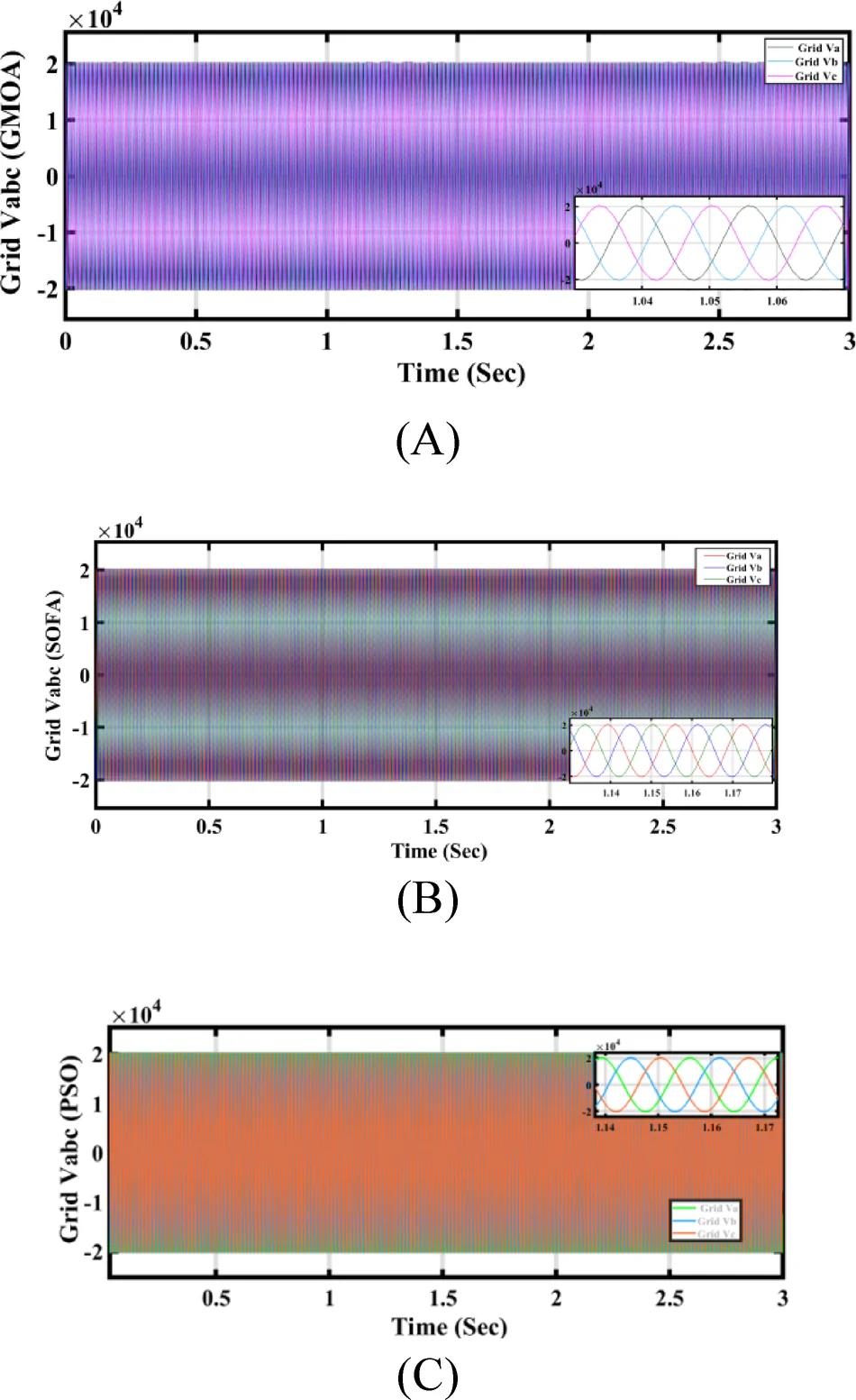
Three-phase grid voltage waveform (Vabc) under PI controller tuning using: (**A**) GMOA, ASFOA and (**C**) PSO.

Figure 39a, b and c specifically illustrate the grid voltage characteristics of solar and wind energy across diverse weather conditions. This investigation focuses on the GMOA, PSO and ASFOA optimization algorithms, with the objective of assessing their performance. The results indicate that the GMOA and PSO algorithms exhibits significant voltage instability, thereby suggesting its inadequacy in maintaining suitable regulation amidst fluctuating environmental influences. Conversely, the ASFOA's grid voltage is notably stable, thereby highlighting its enhanced capacity to respond to abrupt variations in solar irradiance, temperature, and wind velocity. This stability underscores ASFOA's capability in providing a dependable and continuous electricity supply, consequently improving the overall performance and robustness of the integrated renewable energy system.

**Fig. 40:**
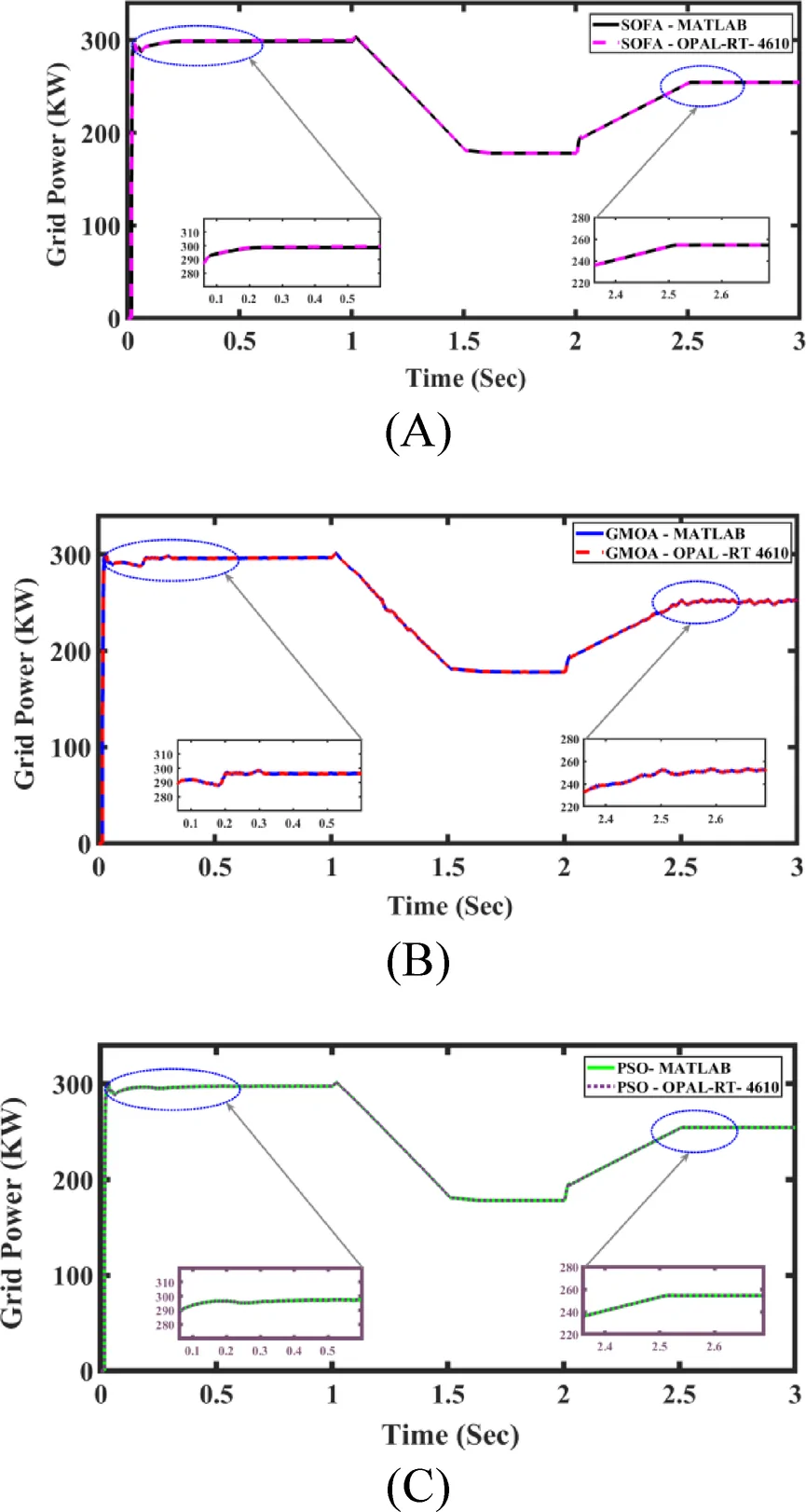
Grid power performance comparison between GMOA, ASFOA and PSO optimization using MATLAB Simulink and OPAL-RT 4610.

As shown in Fig. 40 a, b and c, the grid power results obtained from MATLAB simulations were compared with the experimental outcomes from the OPAL-RT real-time platform for both ASFOA, GMOA and PSO optimization methods. The comparison shows excellent agreement between the three sets of results, indicating that the MATLAB simulations accurately represent the real-time system behavior. Both ASFOA, GMOA and PSO exhibit consistent trends in power generation, with ASFOA maintaining higher precision and stability than GMOA and PSO. This confirms the validity of the simulation models and the reliability of the proposed optimization strategies under dynamic weather conditions.

### Random profile for solar irradiance and wind speed profile

**Fig. 41:**
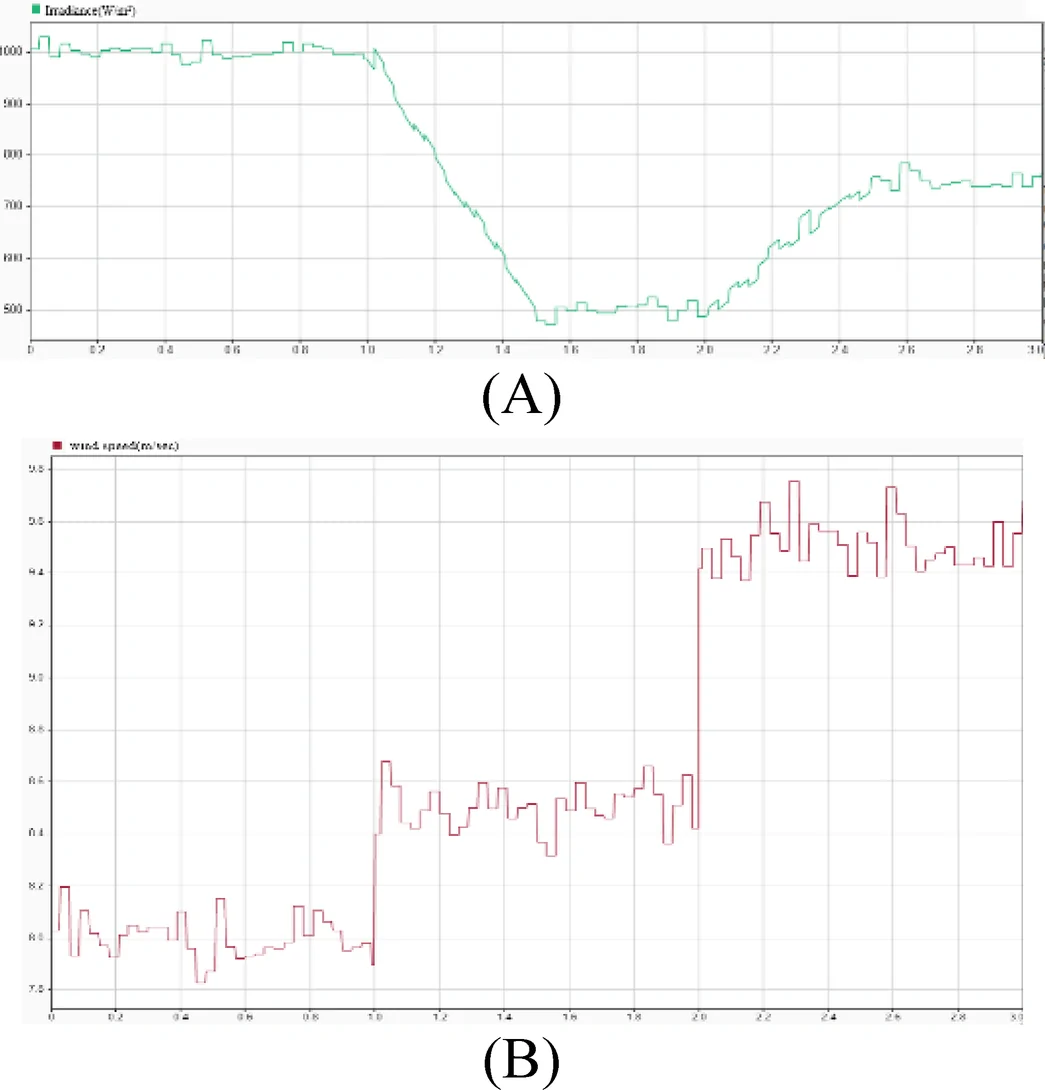
(**A**) Random solar irradiance profile used in the OPAL-RT 4610, (**B**) Random wind speed profile under OPAL-RT 4610.

**Fig. 42:**
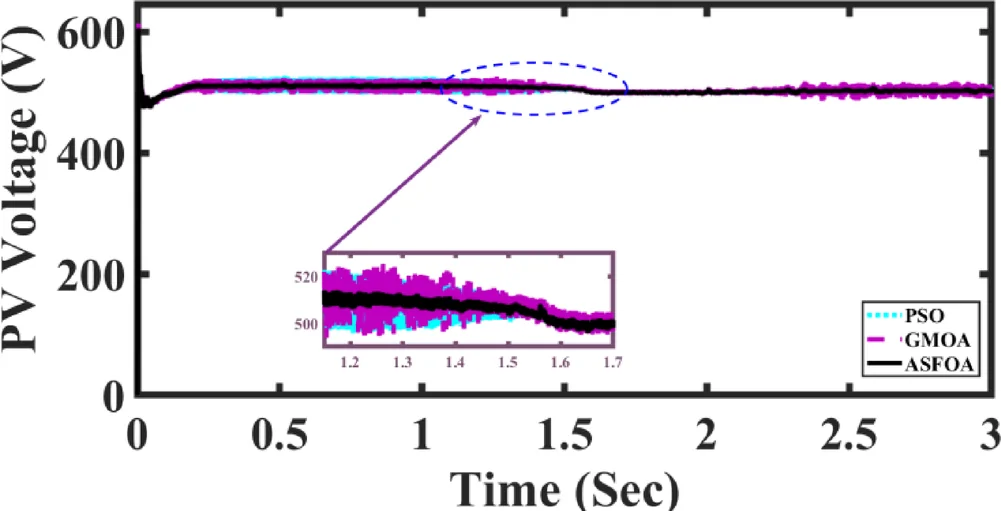
PV output voltage response to random solar irradiance variations validated using the OPAL-RT real-time simulator.

**Fig. 43:**
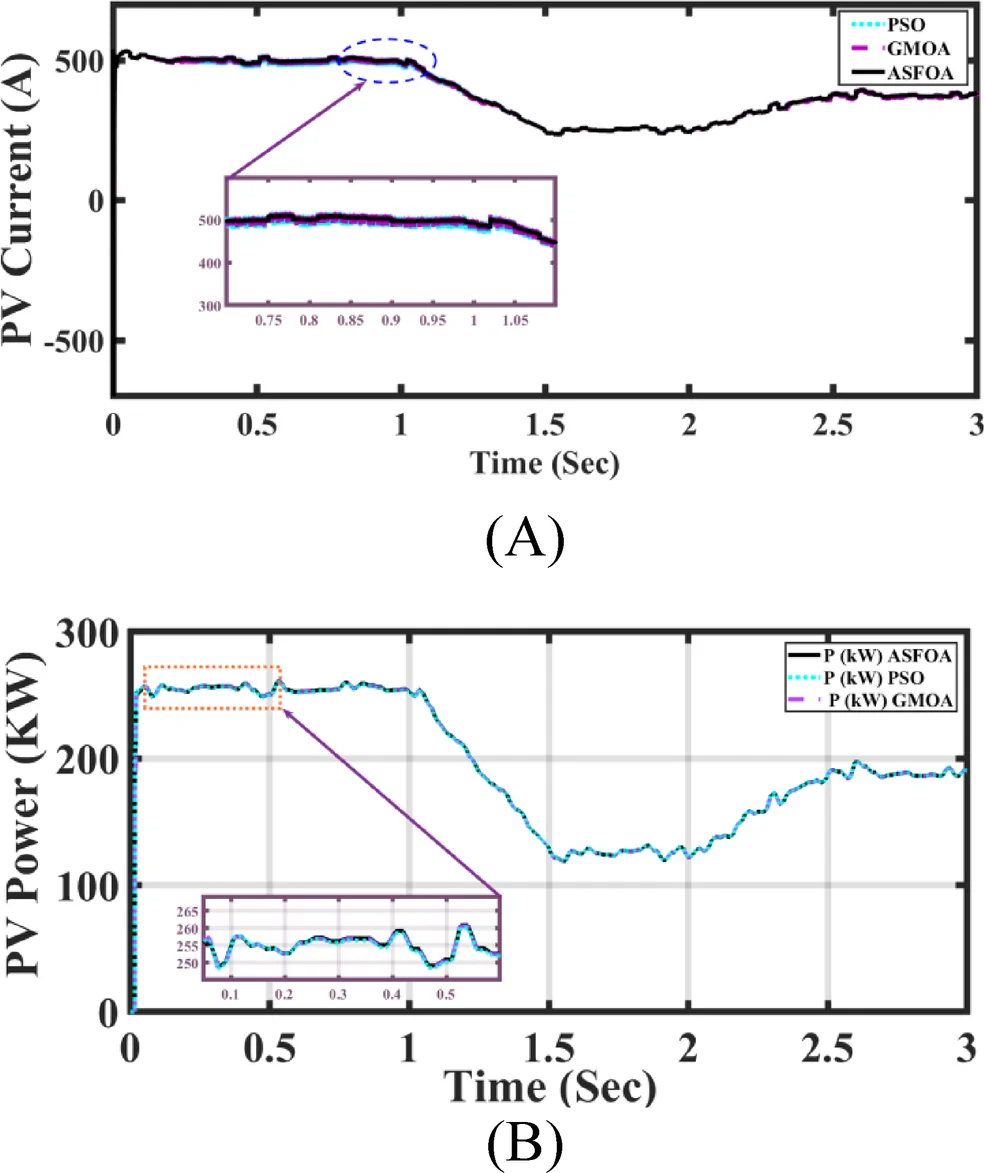
(**A**) PV current and (**B**) PV power performance comparison of the proposed control techniques validated using OPAL-RT real-time simulator.

Figure 41 a, b represents the random variation in both wind speed and solar irradiance applied to the hybrid PV–Wind system during the simulation period. These variations are introduced to emulate realistic environmental conditions and to evaluate the controller’s capability to track the maximum power point under dynamic operating scenarios. The PV output voltage corresponding to the GMOA, PSO and ASFOA control strategies is presented in Fig. 42. It can be observed that the ASFOA -based controller achieves superior voltage regulation compared with the GMOA and PSO approaches. The ASFOA approach produces a more consistent and higher PV output voltage, especially in high-temperature conditions. ASFOA further speeds up the PV system's dynamic response, allowing the controller adapt to changing operating conditions. OPAL-RT real-time platform was used to implement the proposed control technique to verify simulation results. The simulation and experimental results matched well. This proves that ASFOA improves PV system dynamic performance and voltage stability. Figure 43 a and b show the proposed control techniques' PV current and power performance. The results show that the ASFOA-based controller monitors better than GMOA and PSO. ASFOA-operated PV systems have more reliable power extraction and smoother current behavior under various operating conditions. By responding faster and reducing fluctuations, the controller stabilizes the PV system.

The proposed control techniques were implemented on the OPAL-RT real-time platform to further validate the simulation outcomes. The results demonstrated that the ASFOA technique was effective and dependable in making PV current more stable and increasing power production.

**Fig. 44:**
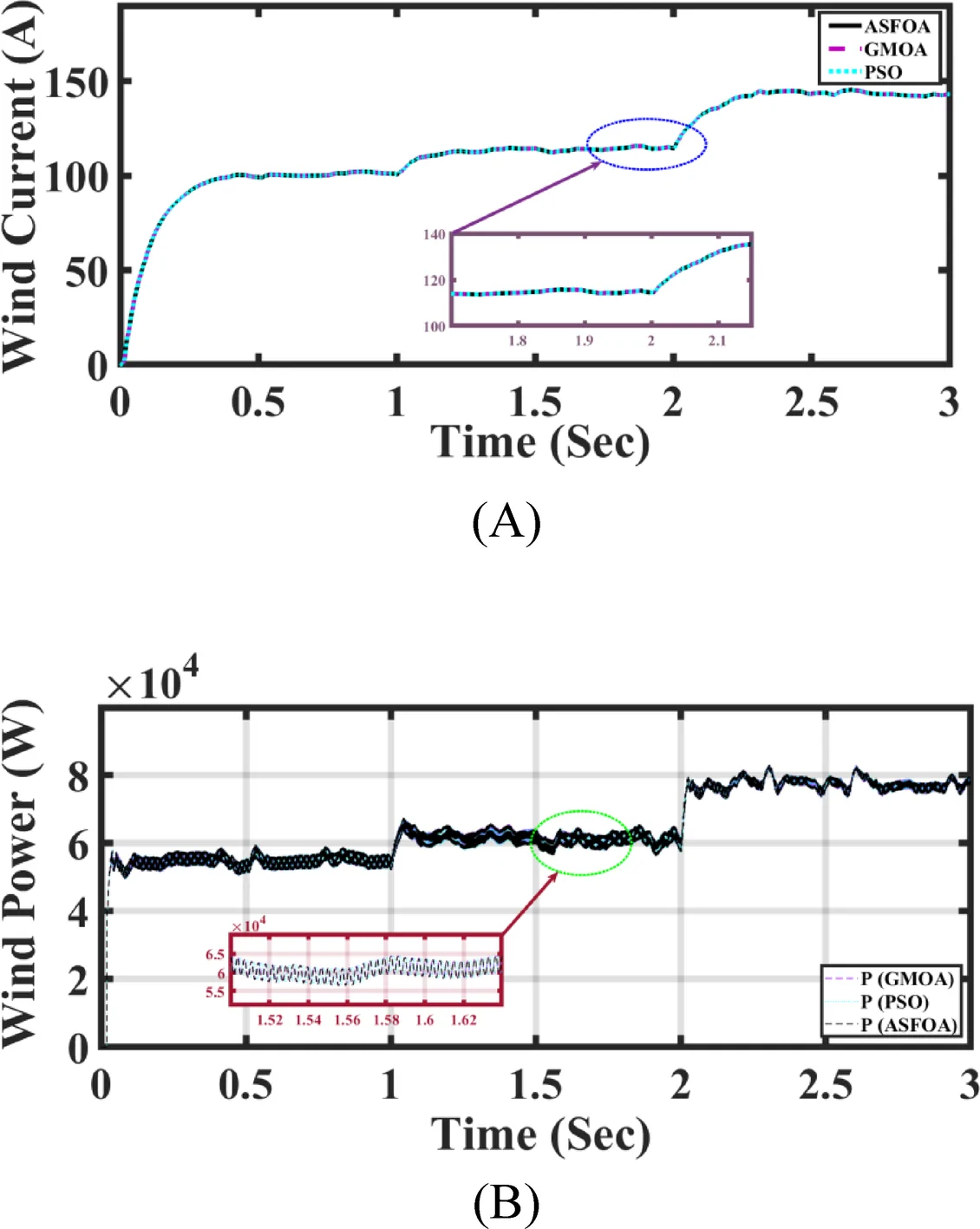
(**A**) Wind current and (**B**) Wind power performance comparison of the proposed control techniques validated using the OPAL-RT real-time simulator.

Figure 44A shows the wind current response, while Fig. 44B displays the wind power extracted by the MPPT controller from the wind turbine. The results demonstrate that the ASFOA-based controller outperforms the GMOA and PSO method in terms of tracking accuracy and reduced oscillations. The enhanced performance stems from the ASFOA's capacity to dynamically adjust controller parameters, thereby facilitating a more precise reaction to fluctuations in wind speed (WS) and promoting smoother system operation. To validate these findings, the proposed control strategies were evaluated on the OPAL-RT real-time platform; the experimental results demonstrated superior performance by the ASFOA, both in terms of response time and power extraction efficiency.

**Fig. 45:**
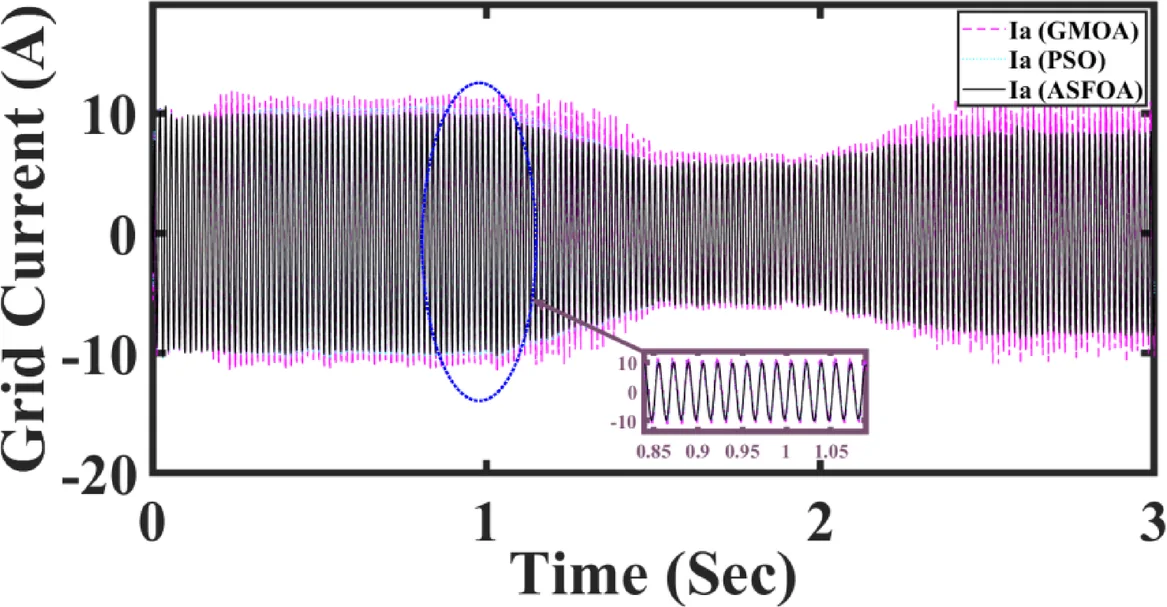
Dynamic response of grid phase current (I_a_) under GMOA, ASFOA and PSO—using OPAL-RT 4610.

Figure 45 depicts the grid current's behavior under varying weather conditions related to solar and wind energy, highlighting the performance assessment of three optimization algorithms: GMOA, PSO and ASFOA. The results demonstrate that the SFOA-based controller offers superior accuracy and stability in current grid regulation compared to the GMOA and PSO technique, hence improving the overall reliability of the integrated renewable energy system. To further substantiate these results, the proposed control techniques were executed and evaluated using the OPAL-RT real-time platform, where the experimental results corroborated the enhanced efficacy of ASFOA in preserving grid current stability amid dynamic environmental conditions.

**Fig. 46:**
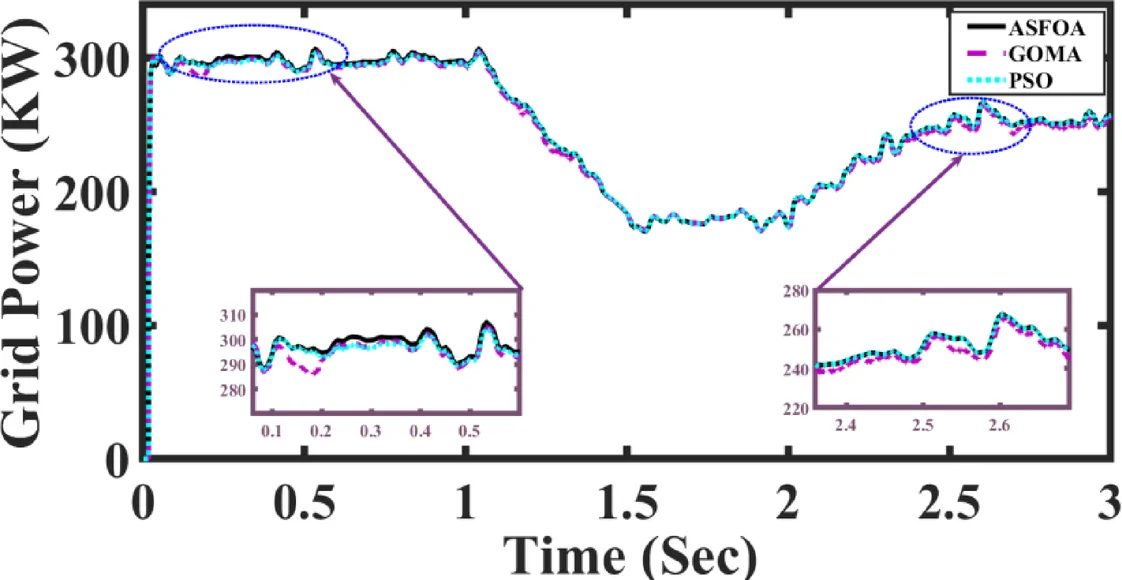
Grid power performance comparison between GMOA, ASFOA and PSO optimization implemented on the OPAL-RT 4610.

**Fig. 47:**
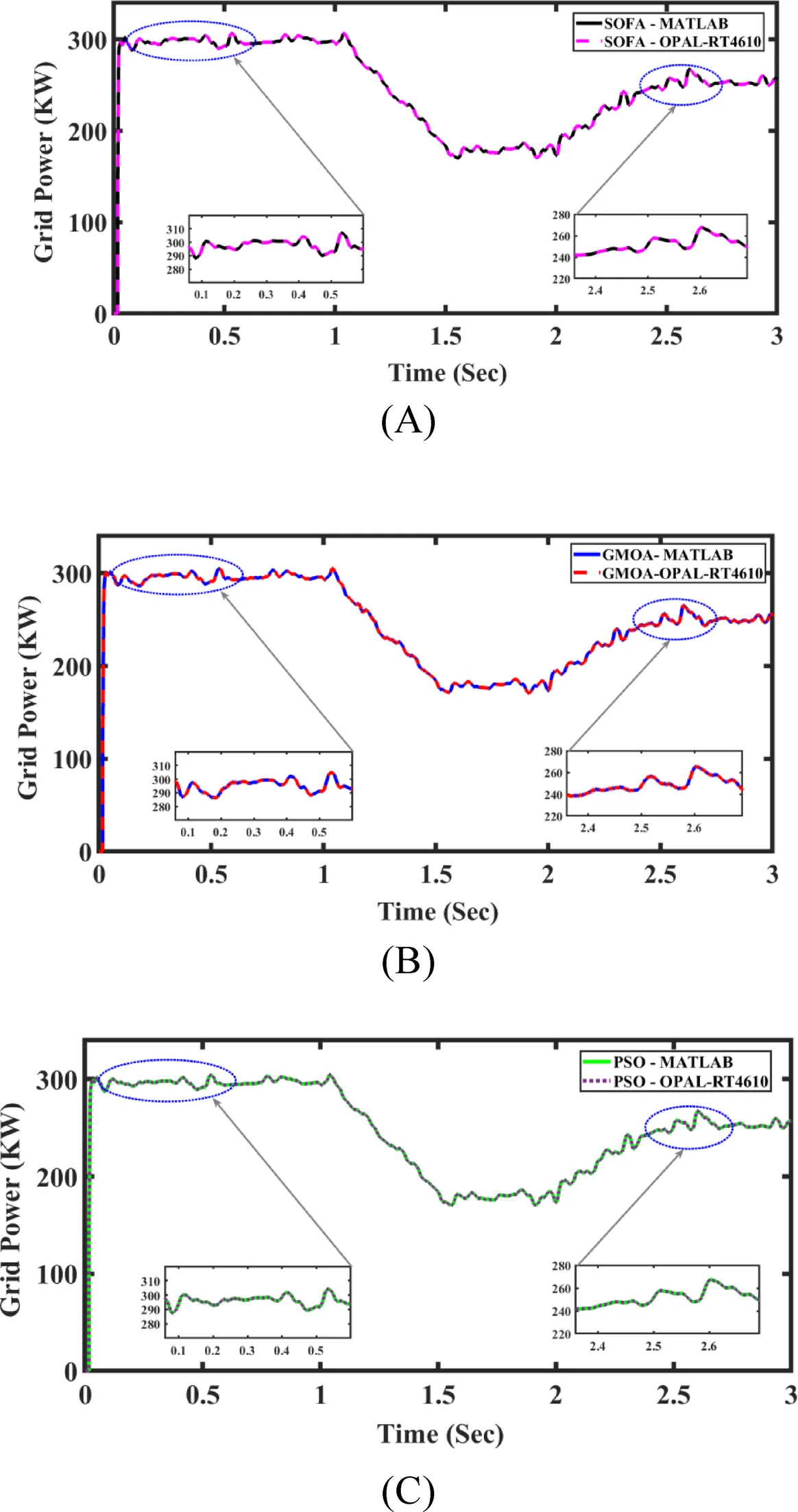
Grid power performance comparison between GMOA, ASFOA and PSO optimization using MATLAB Simulink and OPAL-RT 4610.

A comparison of the GMOA, PSO and ASFOA optimization methodologies' performance is shown in Figure 46, which shows the grid power response under different solar and wind circumstances. When comparing ASFOA, GMOA and PSO, the results show that the former provides better performance in terms of accurate and steady control of grid power. This is proof that ASFOA can keep the power on reliably and consistently, no matter how crazy the weather gets. Through implementation on the OPAL-RT real-time platform, experimental observations confirmed that ASFOA is successful and robust in maintaining exact grid power control, further validating the simulation results. Figure 47A, B and C compare the grid power results for an ASFOA, GMOA and PSO from MATLAB simulations to those from OPAL-RT in real-time. The outcomes demonstrate a high level of concordance, which validates the simulation models' and optimization methodologies' veracity and precision.

**Fig. 48:**
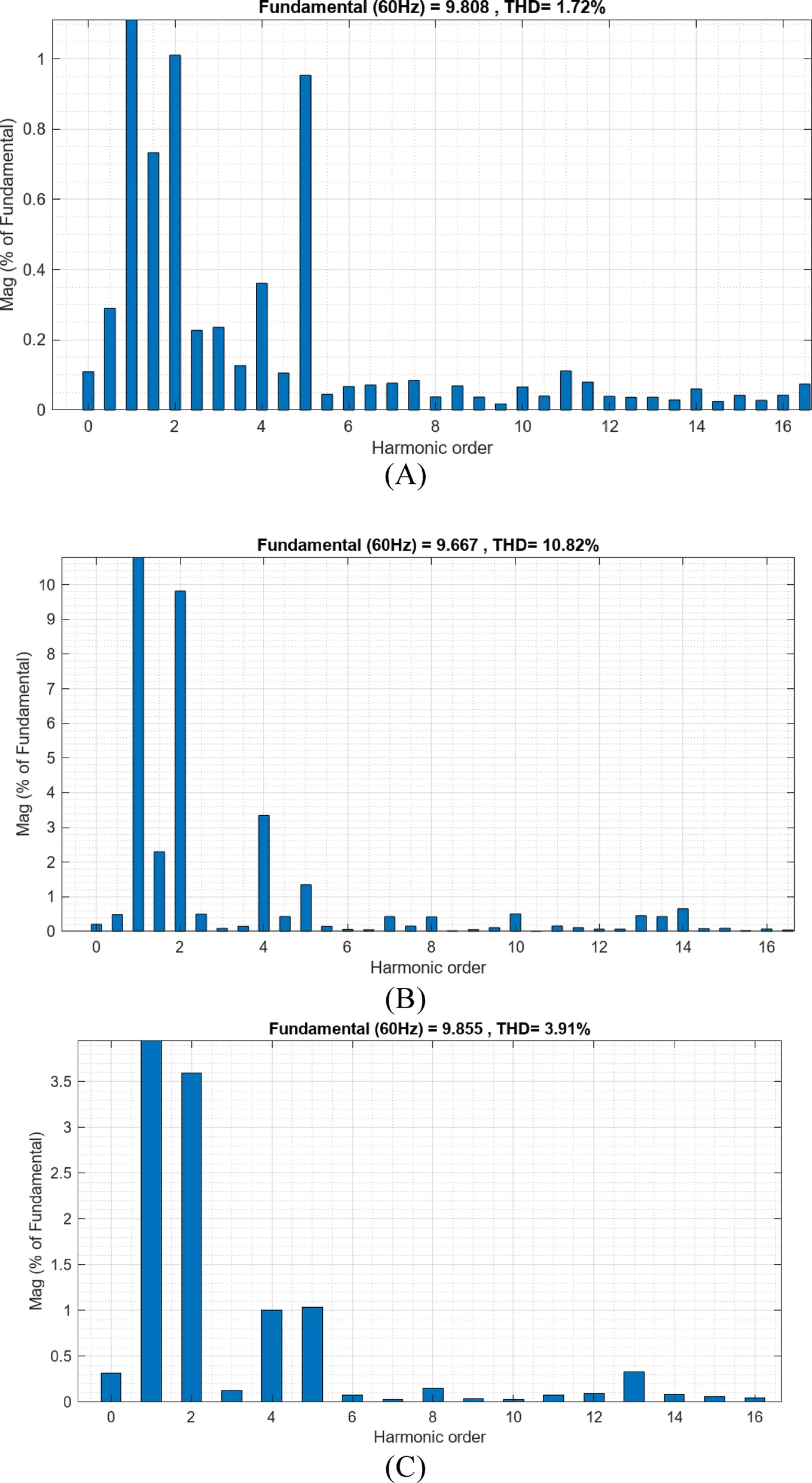
Current THD by using (**A**) ASFOA, (**B**) GOMA and (**C**) PSO.

Figure 48A, B and C, represents the current total harmonics distortion of random variation in both wind speed and solar irradiance applied to the hybrid PV–Wind system during the simulation period. The results indicate that the current THD percentage in the ASOFA technique is lower compared to the GOMA and PSO techniques. This reduction in harmony reflects an improvement in the quality of the output waveform and enhances the overall performance of the system. Lower harmonic content leads to reduced power losses, improved efficiency, and better compatibility with electrical standards. Therefore, the proposed technique demonstrates superior performance in minimizing harmonics and ensuring stable and reliable operation.

**Fig. 49:**
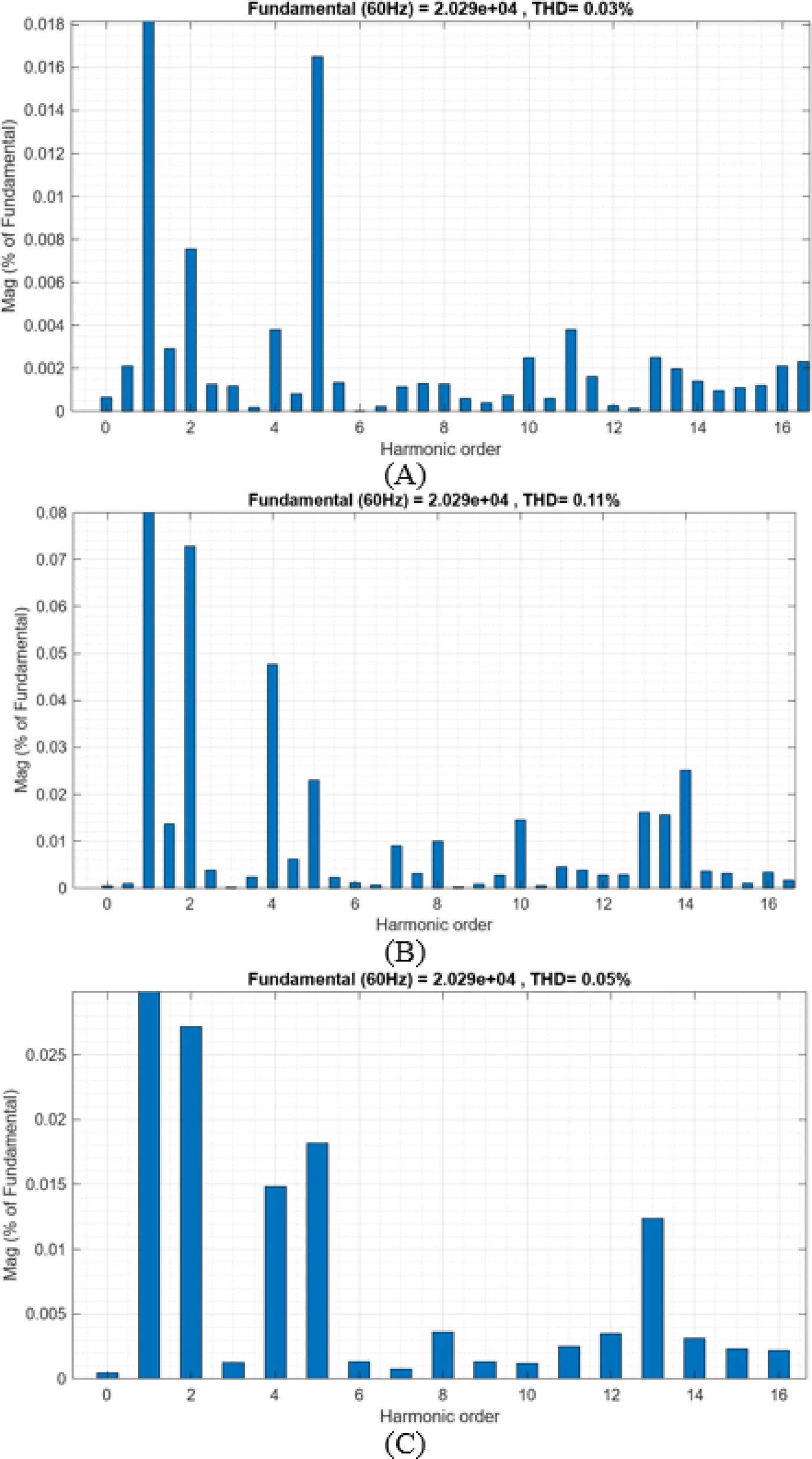
Voltage THD by using (**A**) ASFOA, (**B**) GOMA and (**C**) PSO.

Figure 49A, B and C represents the current total harmonics distortion. The results show that the voltage THD obtained using the ASFOA is lower compared to the results achieved using the GOMA and PSO techniques. This reduction in voltage harmonics indicates a significant improvement in the quality of the output voltage waveform. Lower THD leads to better power quality, enhanced system stability. Therefore, the results confirm that the ASFOA-based approach provides better performance and more effective harmonic mitigation than the GOMA and PSO methods.

The Table 5 summarizes the key performance characteristics, including current and voltage THD, settling time, maximum overshoot, oscillation level ripple, tracking error, dc-link voltage regulation, and MPPT efficiency. this addition complements the graphical results and provides a clearer overall comparison between the three optimization techniques.

**Table 5:** Performance comparison of the GMOA, ASFOA and PSO -based PI controllers.

Performance index	ASFOA	GOMA	PSO
Current THD (%)	1.72	10.82	3.91
Voltage THD (%)	0.03	0.11	0.05
Settling time (s)	0.18	0.24	0.27
Maximum overshoot (%)	0.98	1.96	2.55
Oscillation level ripple (%)	1.57	3.92	5.10
DC-Link voltage regulation (%)	0.20	0.78	1.18
MPPT efficiency (%)	99.8	99.5	98.67
Tracking error (%)	0.17	1.33	1.00

## Conclusions

This study investigated the dynamic performance of a grid-connected hybrid PV–wind energy system under both structured (ramp and step) and random environmental variations, including solar irradiance, temperature, and wind speed. Three optimization-based control strategies, namely the GMOA, PSO and the ASFOA, were employed to determine the optimal PI controller gains and evaluate their effectiveness in enhancing the overall system performance. The results obtained demonstrate that the ASFOA-based controller consistently outperforms the GMOA and PSO-based controller under all investigated operating conditions. Under ramp and step environmental profiles, ASFOA provided faster dynamic response, improved tracking accuracy, and more stable PV voltage and current characteristics, resulting in enhanced maximum power extraction. Similarly, the optimized PI controller achieved superior regulation of the wind turbine rotor speed and output power with reduced oscillations during transient operating conditions. Under random variations in solar irradiance and wind speed, the proposed ASFOA-based controller maintained more stable DC-link voltage regulation, improved grid synchronization, and more reliable active power injection into the utility grid. The comparative analysis indicates that ASFOA increased the grid-injected power by approximately 3.0–4.1% under structured operating conditions and by about 3.3–4.3% under random environmental conditions. In addition, the optimized controller produced lower THD, leading to improved output waveform quality, enhanced current stability, and better overall power quality. These results confirm that optimizing the PI controller gains using advanced metaheuristic algorithms has a direct impact on improving the dynamic behavior and operational efficiency of grid-connected hybrid renewable energy systems. Rather than introducing a new MPPT algorithm, the proposed approach focuses on intelligent controller gain optimization, which consequently enhances maximum power extraction, voltage regulation, and grid stability. From a practical perspective, the proposed optimization framework can be readily integrated into existing grid-connected PV–wind installations without requiring additional hardware modifications. By providing optimal PI controller parameters, the proposed ASFOA-based approach improves controller performance under rapidly changing environmental conditions while maintaining high power quality and reliable grid operation. Furthermore, the successful implementation and validation using the OPAL-RT real-time simulator demonstrates the practicality and feasibility of deploying the proposed control strategy in real-world renewable energy applications. Therefore, the proposed method represents an effective and reliable solution for enhancing the performance, stability, and operational efficiency of future smart renewable energy systems. Future work will focus on extending the proposed optimization framework to multi-objective controller design, incorporating energy storage systems, and investigating adaptive intelligent control strategies for large-scale hybrid renewable energy systems operating under more complex grid conditions.

## Data Availability

All data generated or analyzed during this study are included in this published article.
